# Prevalence, time trends, and correlates of major depressive episode and other psychiatric conditions among young people amid major social unrest and COVID-19 in Hong Kong: a representative epidemiological study from 2019 to 2022

**DOI:** 10.1016/j.lanwpc.2023.100881

**Published:** 2023-08-19

**Authors:** Stephanie M.Y. Wong, Eric Y.H. Chen, Y.N. Suen, Corine S.M. Wong, W.C. Chang, Sherry K.W. Chan, Patrick D. McGorry, Craig Morgan, Jim van Os, David McDaid, Peter B. Jones, T.H. Lam, Linda C.W. Lam, Edwin H.M. Lee, Eric Y.H. Tang, Charlie H. Ip, Winky W.K. Ho, Sarah M. McGhee, P.C. Sham, Christy L.M. Hui

**Affiliations:** a Department of Psychiatry, School of Clinical Medicine, LKS Faculty of Medicine, The University of Hong Kong, Hong Kong SAR, China; b The State Key Laboratory of Brain and Cognitive Sciences, The University of Hong Kong, Hong Kong SAR, China; c School of Public Health, LKS Faculty of Medicine, The University of Hong Kong, Hong Kong SAR, China; d Centre for Youth Mental Health, University of Melbourne, Parkville, VIC, Australia; e Health Service & Population Research Department, Institute of Psychiatry, Psychology, and Neuroscience, King's College London, London, United Kingdom; f Department of Psychiatry and Psychology, Maastricht University Medical Centre, Maastricht, the Netherlands; g Care Policy and Evaluation Centre, Department of Health Policy, London School of Economics and Political Science, United Kingdom; h Department of Psychiatry, University of Cambridge, Cambridge, United Kingdom; i Department of Psychiatry, The Chinese University of Hong Kong, Hong Kong SAR, China

**Keywords:** Major depressive episode, Epidemiological study, Youth mental health, Stressful life events, Social unrest, COVID-19, Environmental stressors

## Abstract

**Background:**

Hong Kong is among the many populations that has experienced the combined impacts of social unrest and the COVID-19 pandemic. Despite concerns about further deteriorations in youth mental health globally, few epidemiological studies have been conducted to examine the prevalence and correlates of major depressive episode (MDE) and other major psychiatric disorders across periods of population-level changes using diagnostic interviews.

**Methods:**

We conducted a territory-wide household-based epidemiological study from 2019 to 2022 targeting young people aged 15–24 years. MDE, generalised anxiety disorder (GAD), panic disorder (PD), and bipolar disorder (BD) were assessed using the Composite International Diagnostic Interview–Screening Scales in 3340 young people. Psychotic disorders were assessed by experienced psychiatrists according to the DSM. Help-seeking patterns were also explored.

**Findings:**

16.6% had any mental disorder (13.7% 12-month MDE, 2.3% BD, 2.1% GAD, 1.0% PD, 0.6% psychotic disorder). The prevalence of MDE increased from 13.2% during period 1 (May 2019–June 2020) to 18.1% during period 2 (July–December 2020), followed by 14.0% during period 3 (January–June 2021) and 13.2% during period 4 (July 2021–June 2022). Different stressors uniquely contributed to MDE across periods: social unrest-related stressors during period 1, COVID-19 stressors during period 2, and personal stressors during periods 3–4. Lower resilience, loneliness, frequent nightmares, and childhood adversity were consistently associated with MDE. Compared to other conditions, those with MDE showed the lowest service utilisation rate (16.7%). Perceiving services to “cost too much” and “talked to friends or relatives instead” were among the major reasons for not seeking help. MDE was also significantly associated with poorer functioning and health-related quality of life.

**Interpretation:**

MDE can be sensitive to population-level changes, although its persistently elevated prevalence across the study period is of concern. Efforts to mitigate their impacts on youth mental health alongside personal risk factors are needed. Further work is required to increase the availability and acceptability of youth-targeted mental health services.

**Funding:**

10.13039/501100005407Food and Health Bureau (HKSAR Government).

**Research in context**

**Evidence before this study**

To review prior evidence in the literature, we first took reference from a large-scale meta-analysis published in 2021 examining the global prevalence of depressive and anxiety disorders due to the COVID-19 pandemic. Of the 48 studies (published between January 1, 2020 and January 29, 2021) that were included in the meta-analysis, only three utilised diagnostic measures. All three of these studies were conducted in adult samples (aged 18 years or above) and in Europe (Czech Republic, Norway, and Spain), with one study having been retracted in 2023. Based on which, we conducted a further systematic search on PubMed for articles published between January 29, 2021 and April 4, 2023 using the following search terms: "mental health", "mental disorders", "depress∗", "depressive disorder” and "prevalence". The search returned 24 results, of which only two studies were conducted in young people, with both studies having been conducted among Chinese college students using self-administered depression and anxiety measures through a web-based survey. PsyArXiv and Google Scholar were additionally searched for preprints and grey literature, which also revealed a lack of representative epidemiological studies focused on young people conducted during this period using interviewer-rated diagnostic measures of mental disorders. No study was found to have examined the prevalence of mental disorders in young people using an epidemiological study design amid multiple population-level stressors, such as social unrest and COVID-19.

**Added value of this study**

This study is the first to examine the prevalence of major depressive episode and other major mental disorders in a representative epidemiological sample of young people using a stratified cluster sampling design. The use of standard diagnostic interviews provides a more accurate prevalence estimate of mental disorders in young people, which adds to previous findings that are predominantly based on self-administered screening measures. This is also the first to examine the associated factors of major depressive episode across periods of large-scale population-level stressors. The examination of mental disorder prevalence across periods of multiple large-scale population-level stressors helps shed light on the combined influences of both individual and external influences on mental health.

**Implications of all the available evidence**

The prevalence and associated factors of mental disorders in young people are related to not only personal but also large-scale changes in the environment, such as major social unrest and COVID-19. As in prior work, the rate of help-seeking is suboptimal, particularly for those with major depressive episode and bipolar disorder. With studies reporting a deteriorating trend of youth mental health even before the COVID-19 pandemic, joint efforts in the development of youth-friendly and effective early intervention programmes and strategies to mitigate the impact of population-level stressors should be prioritised.

## Introduction

The youth period is characterised by immense neurobiological, psychosocial, and developmental changes. The majority of mental disorders, however, also tend to emerge during this period.^1^ The co-occurrence of large-scale societal and global changes over recent years, such as social unrest and the COVID-19 pandemic, has posed even greater mental health challenges.^2^, ^3^, ^4^ With the burden of youth mental disorders and their long-term implications, identifying factors associated with elevated risks related to environmental changes and help-seeking is critical for improving early risk detection and reducing treatment delay.

Across mental disorders, major depression is among the most prevalent and significantly contributes to disability worldwide.^5^^,^^6^ Recent data have shown increased rates of affective and depressive disorders in young people (e.g., 6.3% in 2007 to 13.6% in 2021–2022 for 12-month affective disorders among those aged 16–24 years in Australia^7^; 13.2% in 2017 to 15.8% in 2019 for major depressive episode [MDE] among those aged 12–17 years in the United States^8^^,^^9^). Help-seeking, however, remains suboptimal in this age group.^10^^,^^11^

Despite increasing attention on youth mental health globally, disproportionately few epidemiological studies on the topic have been conducted in the Asian context. There is evidence from studies using self-administered measures suggesting deteriorated mental health in Asian youth samples,^12^ although most of these studies were conducted on student or online samples. Of those that assessed youth mental disorders using diagnostic tools, the focus had often been on those above the age of 18.^10^^,^^13^ To our knowledge, no population-representative epidemiological study of mental health has been conducted in Asia targeting young people aged 15–24 years using standardised diagnostic instruments.

Furthermore, there is a paucity of epidemiological studies conducted amid ongoing waves of population stress which examine potential variations in the presentation of mental disorders and their related factors across periods. An abundance of work has already suggested worsened mental health across populations since the emergence of the COVID-19 pandemic, particularly depressive and anxiety symptoms in young people, although the majority had utilised self-reported or parent-reported measures.^3^ Studies exploring trends in its influences on mental health over time are limited, particularly as the initial waves of COVID-19 subside.^14^^,^^15^

Notably, the population of Hong Kong had additionally experienced an unprecedented series of city-wide social unrest (June 2019–early 2020) before the local outbreak of COVID-19 (January 2020–early 2023).^16^ The ongoing occurrences of large-scale roadblocks, as well as the deployment of tear gas, bean bag rounds, and rubber bullets had caused significant disruption to the everyday lives of the population (e.g., suspension of railway services, tunnels, and school closure).^16^^,^^17^ A previous large family cohort study in Hong Kong has shown the impact of the social unrest on probable depressive symptoms (Patient Health Questionnaire ≥10) among those aged 18 years or above.^4^ Various local community-based studies have also found significant additive influences of social unrest, the initial period of COVID-19 (up to March 2020), and personal life events on depressive symptoms, particularly in young people.^16^^,^^18^, ^19^, ^20^ With the increasing occurrences of social and public health threats globally, a systematic investigation into the prevalence of major mental disorders across periods in the youth population and their associated factors could serve as a reference for future studies, interventions, and policy design in other societies.

Using data from the Hong Kong Youth Epidemiological Study of Mental Health (HK-YES), we aimed to answer several key questions: (1) What is the prevalence of mental disorders in the youth population and how does this compare with other regions? (2) How does the prevalence of mental disorders, specifically MDE, change over time amid population-level changes? (3) How are different factors associated with MDE amid these changes? (4) How is MDE associated with functioning and health-related quality of life (QoL) in young people? And (5) How does help-seeking differ across conditions and what drives help-seeking (or lack of help-seeking)?

## Methods

### Population and study design

Data were from the HK-YES, which is to date the first territory-wide, household-based epidemiological study of youth mental health in Hong Kong. A stratified cluster sampling design was adopted to improve sample representativeness. Invitation letters were posted to a random selection of addresses provided by the Census and Statistics Department of the local government expected to include a young person aged 15–24 years (based on data from the 2016 Population By-Census), stratified by geographic district and housing type. All local residents aged 15–24 years at time of recruitment who consented to the study were eligible. If more than one eligible youth resided in the household, the one whose birthday was closest to the recruitment date was recruited. Consenting participants were invited for a face-to-face interview by a trained researcher with an option of online video conferencing following the same procedures during the pandemic. Study details are described elsewhere,^2^^,^^21^, ^22^, ^23^, ^24^ with details of the sample size calculation provided in Appendix pp 2.

During the recruitment period (May 2019–July 2022), some addresses were invalid (post not deliverable due to address being incorrect, non-existent, or vacant), while 14.6% of verifiable addresses (not returned by the post office) no longer had an eligible youth in the household. Among those contactable, 3460 provided consent (66.5% response rate). This response rate is comparable to those in other epidemiological studies,^25^^,^^26^ with 3340 (96.5%) completing the clinical assessment for mental disorders. Further information regarding potential correlates was available from 3030 participants (90.7%). Participants in the subsample (n = 3030) were slightly older than those not in this subsample (n = 310) (mean = 19.85 [SD = 2.78] years *vs* 19.50 [2.90]), *p* = 0.042, while no significant differences in sex, psychiatric history, and prevalence of any mental disorder were observed, *p* > 0.05. While all participants were aged 15–24 years at recruitment, eight participants (0.2%) were 25 years at the time of assessment. Since some studies also considered the youth period to persist up to about the age of^25^^,^^27^ these participants were also included in the present study.

Informed consent was obtained from all participants. For those below the age of 18 years, consent was obtained both from participants and their parents/guardians. Ethics approval was granted by the Institutional Review Board of the University of Hong Kong/Hospital Authority Hong Kong West Cluster. The STROBE reporting guideline was followed in this study.

### Key measures

Past 12-month major depressive episode (MDE) was assessed using the interviewer-rated WHO Composite International Diagnostic Interview–Screening Scales (CIDI-SC).^28^ The CIDI-SC is a fully-structured lay-administered instrument that assesses a range of mental disorders according to the DSM-IV criteria embedded in the CIDI. The instrument has been adopted in other large-scale studies and has been validated for use in adolescents, including in the WHO World Mental Health International College Student Initiative.^29^^,^^30^ MDE is defined as the experience of five or more of the following core symptoms of depression for most of the day, nearly every day, during the same two-week period: (i) depressed mood; (ii) diminished interest/pleasure in activities; (iii) significant change in weight or appetite; (iv) insomnia/hypersomnia; (v) psychomotor agitation/retardation; (vi) fatigue; (vii) feelings of worthlessness or excessive guilt; (viii) difficulties in thinking/concentrating; and (ix) suicidal ideation, plan, or attempt. Depressed mood or diminished interest/pleasure in activities, and significant distress or impairment as a result of these symptoms, were required for establishing the presence of MDE.

Aside from MDE, generalised anxiety disorder (GAD), panic disorder (PD), and bipolar disorder (BD; including mania or hypomania) were also assessed using the CIDI-SC. Lifetime and 12-month prevalence of each disorder were evaluated, with past 30-day prevalence also evaluated for MDE and GAD. Psychotic disorder was assessed via a two-stage procedure, wherein those endorsing one or more items on the CIDI-3.0 psychosis screen, having an existing diagnosis, or having received antipsychotic medication were further interviewed by experienced team psychiatrists according to the Structured Clinical Interview for DSM. Any mental disorder was defined as presence of 12-month MDE, GAD, PD, BD, or psychotic disorder.

Functioning was assessed using the interviewer-rated Social and Occupational Functioning Assessment Scale (SOFAS)^31^ and total days (past 30 days) with reduced and lost productivity due to mental distress, respectively. Health-related QoL was assessed using the EuroQol-5D (EQ-5D-5L)^32^ and the 12-Item Short Form Survey (SF-12),^33^ the latter consisting of two subscales reflecting physical and mental health-related QoL. Global mental well-being was assessed using the World Health Organization–Five Well-Being Index (WHO-5).^34^

Services utilised for mental health reasons at time of interview and over lifetime were assessed using a checklist consisting of psychiatrist, psychologist, community psychiatric nurse, general practitioner, non-psychiatric clinical/nursing services, social worker, occupational therapist, traditional Chinese practitioner, religious services, day centre, and others. Reasons for not seeking help were further assessed using items from the CIDI-3.0. Those not receiving any psychiatric/psychological services were asked whether they ever felt a need for counselling or medication during the past 12 months for their emotional problems. Those who reported “yes” were asked to rate the degree to which they considered the following statements to be important reasons for their lack of help-seeking on a 5-point Likert scale (from “unimportant” to “very important”): “unsure if available treatments were very effective”; “wanted to handle the problem on your own”; “too embarrassed”; “talked to friends or relatives instead”; “costs too much money”; “unsure where to go/who to see”; “had problems with time, transportation, or scheduling”; “afraid it might harm your school/professional career”; and “worried people would treat you differently if they knew”.

Measures of personal and environmental risk factors, including background factors (sex, age, any government financial assistance received—as a measure of socioeconomic status, and childhood adversity), psychological factors (including resilience and loneliness), digitalisation and lifestyle (including smartphone overuse, frequent nightmares, and physical activity), and exposure to personal and population-level stressors (including personal stressful life events [SLEs], social unrest-related traumatic events [TEs], and COVID-19 pandemic-related events [PEs]), as well as their validity for use in the present youth sample, are detailed in Supplementary Table S1 (Appendix pp 5).

### Statistical analysis

The prevalence of mental disorders was first estimated with weighting adjustments according to sex and age data of the local Census to approximate demographics of the youth population. As participants aged 15–19 and 20–25 years may face different developmental and psychosocial challenges, we examined potential differences in the prevalence of mental disorders between these age groups.

Since few participants presented conditions apart from MDE, risk factor analyses were conducted only for 12-month MDE in the subsample (n = 3030). Univariate logistic regression was first applied across the entire study period, followed by a multivariable logistic regression using the backward selection method to identify a more parsimonious set of associated factors. The same set of multivariable logistic regression was applied within the two age groups (15–19, 20–25 years). An additional multivariable logistic regression model was applied with all factors significant in the univariate analysis (after accounting for multiple comparison) for ensuring robustness of the findings. Key personal background factors, namely sex, age, and childhood adversity, were included in all models regardless of their significance due to their biological plausibility and confounding influences.

We then explored time trends in 12-month prevalence of MDE, wherein participants were categorised into four groups according to their time of interview. Differences in prevalence across the four periods were tested using *χ*^2^ tests. Separate multivariable models including the same set of variables were applied using the backward selection method within each period. As sensitivity analysis, further analyses were conducted after excluding those with other (non-MDE) disorders to ensure findings were not influenced by comorbid conditions. Although no significant difference was found in the prevalence of any mental disorder between participants with and without missing data, we applied multiple imputation using the open-source software XGBoost to maximise the information collected from participants and to reduce potential bias, similar to prior work.^35^^,^^36^

After examining the associations between MDE and functioning and health-related QoL, service utilisation across each condition, correlates of psychiatric/psychological service use among those with MDE, and reasons for not seeking help were explored. ORs with 95% CIs were presented for all logistic regression models, wherein ORs of continuous predictors reflect the effect on MDE per 1-unit change. Statistical significance was set at the 0.05 level, with multiple comparisons accounted for in univariate analyses. All analyses were undertaken with SPSS (version 27.0) and R.

### Role of the funding source

The funders of the study had no role in the design and conduct of the study, data collection, management, analysis, and interpretation, writing of the manuscript, or decision to submit the manuscript.

## Results

### Prevalence of mental disorders

16.6% of young people had at least one mental disorder (weighted). Most (13.7%) met the criteria for one disorder only, with 2.5% presenting two and 0.3% presenting three or more disorders. MDE was most common (weighted 12-month prevalence: 13.7%), while the prevalence of other disorders was: GAD 2.1%, PD 1.0%, BD 2.3%, and psychotic disorder 0.6% (Table 1). 11.1% met the criteria for only 12-month MDE and not other disorders. The prevalence of MDE with comorbid GAD, PD, BD, and psychotic disorder were 1.4%, 0.7%, 0.7%, and 0.2%, respectively. Although the prevalence of other disorders was considerably lower, significantly more young people with 12-month MDE also met the criteria for other DSM-IV conditions compared to those without MDE, all *p* < 0.001 (Supplementary Figure S1, Appendix pp 3).

**Table 1:** Prevalence of DSM-IV MDE and selected mental disorders in the representative epidemiological youth sample (n = 3340).

DSM-IV disorders	Time frame	HK-YES participants (n = 3340)			
		Unweighted		Weighted	
		%	SE	%	SE
**Any disorder**^a^					
1 disorder only		14.5	0.6	13.7	0.6
≥2 disorders		3.0	0.3	2.8	0.3
**DSM-IV conditions**					
Major depressive episode (MDE)	Lifetime	24.5	0.7	23.4	0.7
	12-month	14.6	0.6	13.7	0.6
	30-day	3.0	0.3	2.9	0.3
Generalised anxiety disorder (GAD)	Lifetime	3.1	0.3	3.1	0.3
	12-month	2.2	0.2	2.1	0.2
	30-day	1.7	0.2	1.7	0.2
Panic disorder (PD)	Lifetime	1.6	0.2	1.4	0.2
	12-month	1.1	0.2	1.0	0.2
Bipolar disorder (BD)	Lifetime	4.2	0.3	4.2	0.4
	12-month	2.3	0.3	2.3	0.2
Psychotic disorder	Lifetime	0.6	0.1	0.6	0.1

Weighting adjustments were applied according to sex and age data of the local Census. HK-YES = the Hong Kong Youth Epidemiological Study of Mental Health.

a Any disorder is defined as the presence of 12-month MDE, GAD, PD, or BD, or psychotic disorder.

Considering potential age effects, those aged 20–25 years showed higher rates of 12-month MDE (15.9%) compared to those aged 15–19 years (12.9%), *p* = 0.015, although the difference was not significant after adjusting for multiple comparisons. No significant age difference in other disorders was observed (Supplementary Figure S2, Appendix pp 4). Participant age was adjusted for in all subsequent multivariable models.

### Factors associated with MDE

Differences in personal risk factors and stress exposure between those with and without 12-month MDE are presented in Supplementary Table S2 (Appendix pp 6). After accounting for multiple comparisons in the univariate analyses, female sex, childhood adversity, lower resilience, higher loneliness, smartphone overuse, frequent nightmares, fewer days of regular exercise, as well as all three types of external stressors, were significantly associated with 12-month MDE (Table 2).

**Table 2:** Univariate and multivariable logistic regression models for 12-month MDE in the epidemiological youth sample.

Variables	Sample (n = 3030)	12-month MDE (n = 436)		Adjusted OR (95% CI)	*p*
		Unadjusted OR (95% CI)	*p*		
**Background factors**					
Male sex		1 [Ref]		1 [Ref]	
Female sex	1763 (58.2%)	**12.01 (1.61–2.51)**	**<0.001**	**1.93 (1.49–2.49)**	**<0.001**
Age	19.85 (2.78)	1.04 (1.01–1.08)	0.044	1.02 (0.98–1.07)	0.37
No childhood adversity		1 [Ref]		1 [Ref]	
Has childhood adversity	1075 (35.5%)	**3.05 (2.48–3.75)**	**<0.001**	**1.89 (1.46–2.43)**	**<0.001**
Not receiving government financial assistance		1 [Ref]		–	–
Receiving government financial assistance^a^	287 (9.5%)	1.09 (0.78–1.53)	0.62	–	–
Born in Hong Kong	2437 (80.4%)	1 [Ref]		–	–
Not born in Hong Kong		1.01 (0.78–1.31)	0.93	–	–
**Psychological factors**					
Resilience (CD-RISC-10)	24.03 (6.27)	**0.89 (0.87–0.90)**	**<0.001**	**0.94 (0.92–0.96)**	**<0.001**
Loneliness (UCLA-LS)	43.91 (9.00)	**1.09 (1.08–1.11)**	**<0.001**	**1.06 (1.05–1.08)**	**<0.001**
**Digitalisation and lifestyle**					
No smartphone overuse (CIAS-R <67)		1 [Ref]		1 [Ref]	
Smartphone overuse (CIAS-R ≥67)	905 (29.9%)	**2.17 (1.76–2.67)**	**<0.001**	**1.28 (1.01–1.64)**	**0.041**
No frequent nightmares (<1/week)		1 [Ref]		1 [Ref]	
Frequent nightmares (≥1/week)	508 (16.8%)	**3.46 (2.76–4.33)**	**<0.001**	**2.20 (1.70–2.87)**	**<0.001**
Days of regular exercise (past week)	1.71 (1.56)	**0.90 (0.84–0.96)**	**0.002**	–	–
**Family relationship**					
Family dysfunction (BFRS)	19.68 (7.15)	**1.09 (1.07–1.10)**	**<0.001**	1.02 (1.00–1.04)	0.067
**Personal and population-level stressors**					
<2 personal SLEs		1 [Ref]		1 [Ref]	
≥2 personal SLEs	674 (22.2%)	**2.00 (1.60–2.49)**	**<0.001**	**1.61 (1.25–2.08)**	**<0.001**
<2 social unrest-related TEs		1 [Ref]		1 [Ref]	
≥2 social unrest-related TEs	576 (19.0%)	**1.47 (1.16–1.87)**	**0.002**	**1.45 (1.09–1.91)**	**0.011**
<2 COVID-19 stressors^b^		1 [Ref]		1 [Ref]	
≥2 COVID-19 stressors^b^	1052 (36.7%)	**1.50 (1.21–1.85)**	**<0.001**	**1.45 (1.09–1.92)**	**0.028**

Data are from 3030 participants of the HK-YES.

Statistics significant after accounting for multiple comparisons in the univariate analysis are boldfaced (0.05/14 = 0.004). The backward selection method was used for the multivariable logistic regression model. Any government assistance received, place of birth, and days of regular exercise were considered but removed from the final multivariable model.

BFRS = Brief Family Relationship Scale; CD-RISC-10 = Connor-Davidson Resilience Scale 10-Item; CIAS-R = Revised Chen Internet Addiction Scale; MDE = major depressive episode; PEs = COVID-19 pandemic-related events; PSQI = Pittsburgh Sleep Quality Index; SLEs = Stressful Life Events; TEs = social unrest-related traumatic events; UCLA-LS = 20-item UCLA Loneliness Scale.

a Data on any government financial assistance received were available from 3024 participants.

b Data on COVID-19 stressors were collected since March 2020 and were available from 2870 participants.

Using the backward selection method, the multivariable logistic regression model showed that female sex (OR = 1.93 [95% CI, 1.49–2.49]), childhood adversity (OR = 1.89 [1.46–2.43]), lower resilience (OR = 0.94 [0.92–0.96]), higher loneliness (OR = 1.06 [1.05–1.08]), smartphone overuse (OR = 1.29 [1.01–1.64]), frequent nightmares (OR = 2.20 [1.70–2.87]), and exposure to personal SLEs (OR = 1.61 [1.25–2.08]), social unrest-related TEs (OR = 1.45 [1.09–1.92]), and COVID-19 PEs (OR = 1.31 [1.03–1.66]) were significantly associated with MDE (Table 2). Any government assistance received, place of birth, and days of regular exercise were also considered but removed from the final model. Findings from the multivariable model based on significance in the univariate analyses were comparable and are presented in Supplementary Table S3 (Appendix pp 7). Similar findings were also observed when those with other disorders (n = 83) were excluded from the model (Supplementary Table S4, Appendix pp 8).

The overall pattern of associations was similar in the age subgroup analyses (Supplementary Table S5, Appendix pp 9). Nevertheless, with all other variables in the multivariable model accounted for, social unrest-related TEs (OR = 1.73 [1.06–2.80]) and smartphone overuse (OR = 1.63 [1.12–2.37]) were associated with MDE only in the younger age group, whereas COVID-19 PEs (OR = 1.53 [1.12–2.09]) and fewer days of exercise (OR = 0.89 [0.80–0.99]) were associated with MDE only in the older age group. Lower resilience, higher loneliness, frequent nightmares, exposure to personal SLEs, as well as female sex and childhood adversity, were associated with MDE in both age groups (see Appendix pp 9).

### Time trends in MDE: 2019 to 2022

**Fig. 1:**
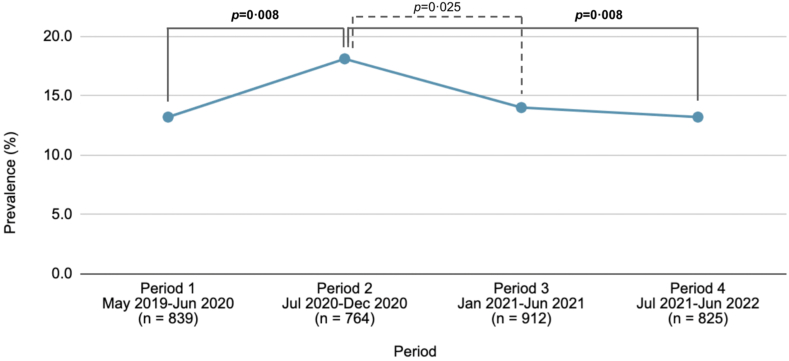
**Trends in the 12-month prevalence of DSM-IV MDE in the epidemiological youth sample in Hong Kong 2019–2022 (n = 3340).** Note: Prevalence data (unweighted) were from the 3340 participants of the HK-YES. Only differences significant at the *p* < 0.05 level are presented. Differences that remained significant after accounting for multiple comparisons (0.05/6 = 0.0083) are in boldface. MDE = major depressive episode.

Across periods from 2019 to 2022, the rate of 12-month MDE significantly increased from 13.2% during period 1–18.1% during period 2, *p* = 0.008 (Fig. 1). This rate subsequently declined to 14.0% during period 3, *p* = 0.025, and 13.2% during period 4, *p* = 0.008 (all unweighted).

#### Factors associated with MDE across periods

Potential changes in the correlates of MDE across the four periods were then examined. Overall, lower resilience, higher loneliness, frequent nightmares, and childhood adversity were consistently associated with MDE across all four periods. Being female was significant through periods 1–3 (Table 3).

**Table 3:** Multivariable logistic regression showing factors associated with 12-month MDE across periods amid ongoing population-level stressors, 2019–2022 (n = 3030).

Variable	12-month MDE			
	Period 1 (May 2019–Jun 2020) (n = 100/739)	Period 2 (Jul 2020–Dec 2020) (n = 126/706)	Period 3 (Jan 2021–Jun 2021) (n = 112/851)	Period 4 (Jul 2021–Jun 2022) (n = 98/734)
	Adjusted OR (95% CI)	Adjusted OR (95% CI)	Adjusted OR (95% CI)	Adjusted OR (95% CI)
Background factors				
Male sex	1 [Ref]	1 [Ref]	1 [Ref]	1 [Ref]
Female sex	**2.14 (1.24–3.71)**∗∗	**1.99 (1.24–3.19)**∗∗	**2.26 (1.36–3.75)**∗∗	1.54 (0.93–2.55)
Age	0.91 (0.83–1.00)	1.06 (0.98–1.15)	1.08 (0.99–1.17)	1.02 (0.94–1.11)
No childhood adversity	1 [Ref]	1 [Ref]	1 [Ref]	1 [Ref]
Has childhood adversity	**2.02 (1.21–3.37)**∗∗	**1.87 (1.20–2.91)**∗∗	**1.66 (1.06–2.61)**∗	**2.27 (1.40–3.68)**∗∗
Not receiving government financial assistance	–	–	–	–
Receiving government financial assistance	–	–	–	–
Born in Hong Kong	–	–	–	–
Not born in Hong Kong	–	–	–	–
Psychological factors				
Resilience (CD-RISC-10)	**0.93 (0.89–0.97)**∗∗∗	**0.96 (0.92–0.99)**∗	**0.90 (0.87–0.94)**∗∗∗	**0.93 (0.90–0.97)**∗∗
Loneliness (UCLA-LS)	**1.07 (1.03–1.11)**∗∗∗	**1.04 (1.02–1.07)**∗∗	**1.06 (1.03–1.09)**∗∗∗	**1.08 (1.05–1.11)**∗∗∗
Lifestyle factors				
No smartphone overuse (CIAS-R<67)	–	1 [Ref]	–	–
Smartphone overuse (CIAS-R≥67)	–	**1.81 (1.16–2.83)**∗∗	–	–
No frequent nightmares (<1/week)	1 [Ref]	1 [Ref]	1 [Ref]	1 [Ref]
Frequent nightmares (≥1/week)	**2.47 (1.49–4.10)**∗∗∗	**2.64 (1.61–4.33)**∗∗∗	**2.30 (1.33–3.95)**∗∗	**2.00 (1.16–3.47)**∗
Days of regular physical activity (past week)	–	**0.85 (0.72–0.99)**∗	–	1.02 (0.86–1.20)
Family functioning				
Poor family functioning (BFRS)	**1.04 (1.01–1.08)**∗	–	–	–
Personal and population-level stressors				
<2 personal SLEs	–	–	1 [Ref]	1 [Ref]
≥2 personal SLEs	–	–	**2.18 (1.30–3.65)**∗∗	**2.05 (1.20–3.50)**∗∗
<2 social unrest-related TEs	1 [Ref]	–	–	–
≥2 social unrest-related TEs	**2.07 (1.25–3.43)**∗∗	–	–	–
<2 COVID-19 PEs	–	1 [Ref]	–	–
≥2 COVID-19 PEs	–	**1.87 (1.18–2.96)**∗∗	–	–

Backward stepwise method was used for the multivariable logistic regression models presented. Key personal background variables, including sex, age, and childhood adversity were included in all analyses regardless of their significance.

Statistics significant at the *p* < 0.05 level are boldfaced.

BFRS = Brief Family Relationship Scale; CD-RISC-10 = Connor-Davidson Resilience Scale 10-Item; CIAS-R = Revised Chen Internet Addiction Scale; MDE = major depressive episode; PEs = COVID-19 pandemic-related events; SLEs = Stressful Life Events; TEs = social unrest-related traumatic events; UCLA-LS = 20-item UCLA Loneliness Scale. ∗*p* < 0.05, ∗∗*p* < 0.01, ∗∗∗*p* < 0.001.

Interestingly, the associations between different stressors and MDE appeared to be specific across periods: only social unrest-related TEs during period 1 (OR = 2.07 [1.25–3.43]), COVID-19 PEs during period 2 (OR = 1.87 [1.18–2.96]), and personal SLEs during periods 3 (OR = 2.18 [1.30–3.65]) and 4 (OR = 2.05 [1.20–3.50]). Family functioning was significant only during period 1 (OR = 1.04 [1.01–1.08]), while smartphone overuse (OR = 1.81 [1.16–2.83]) and fewer days of moderate-to-vigorous physical activity (OR = 0.85 [0.72–0.99]) were significant only during period 2 (Table 3).

Similar findings were observed in the multivariable logistic regression models adjusting for all variables significant in the univariate analyses (Supplementary Table S6, Appendix pp 10) and after excluding those with other disorders (Supplementary Table S7, Appendix pp 11). Notably, findings from the multiple imputation models were also similar to the complete-case analyses (see Supplementary Table S8, Appendix pp 12).

### MDE, functioning, and quality of life

Those with 12-month MDE showed significantly poorer overall functioning (mean = 77.09 [SD = 8.35] *vs* 83.60 [7.19]) and more days of reduced (5.53 [7.35] *vs* 1.63 [3.80]) and lost productivity (1.64 [4.29] *vs* 0.19 [1.31]), all *p* < 0.001. They also showed poorer health-related QoL (EQ-5D-5L: mean = 0.90 [SD = 0.11] *vs* 0.96 [0.07]; PCS-12: 50.38 [8.24] *vs* 52.84 [6.69]; MCS-12: 31.17 [11.77] *vs* 43.10 [10.54]) and mental well-being (WHO-5: 40.64 [17.97] *vs* 56.82 [18.15]), all *p* < 0.001 (Appendix pp 13).

### Service utilisation

73.4% of those with 12-month MDE were not utilising any service for mental health needs (Table 4). Only 16.7% of those with 12-month MDE, and 27.2% of those with 30-day MDE, were utilising psychiatric/psychological services. The rate was similar for BD (16.9%), higher for GAD (28.1%) and PD (43.8%), and highest for psychotic disorders (61.5%) (Table 4). A breakdown of the specific services used across conditions is provided in Supplementary Table S10 (Appendix pp 14).

**Table 4:** Rates of service utilisation across DSM-IV mental disorders in the epidemiological youth sample in Hong Kong (n = 3030).

Time of service use	Service type		
	Psychiatric/Psychological services (%)	Other services only^a^ (%)	None (%)
12-month MDE (n = 436)			
Current	73 (16.7)	43 (9.9)	320 (73.4)
Lifetime	131 (30.0)	124 (28.4)	181 (41.5)
30-day MDE (n = 92)			
Current	25 (27.2)	9 (9.8)	58 (63.0)
Lifetime	35 (38.0)	23 (25.0)	34 (37.0)
12-month GAD (n = 64)			
Current	18 (28.1)	6 (9.4)	40 (62.5)
Lifetime	26 (40.6)	16 (25.0)	22 (34.4)
30-day GAD (n = 50)			
Current	17 (34.0)	5 (10.0)	28 (56.0)
Lifetime	22 (44.0)	12 (24.0)	16 (32.0)
12-month PD (n = 32)			
Current	14 (43.8)	1 (3.1)	17 (53.1)
Lifetime	17 (53.1)	6 (18.8)	9 (28.1)
12-month BD (n = 71)			
Current	12 (16.9)	5 (7.0)	54 (76.1)
Lifetime	20 (28.2)	15 (21.1)	36 (50.7)
Psychotic disorder (n = 13)			
Current	8 (61.5)	1 (7.7)	4 (30.8)
Lifetime	11 (84.6)	1 (7.7)	1 (7.7)

a Other services include those from general practitioners, non-psychiatric clinical/nursing services, social workers, occupational therapists, traditional Chinese practitioners, religious services, day centre, and others.

#### Factors associated with service utilisation among young people with MDE

Among those with 12-month MDE (n = 436), childhood adversity (OR = 1.95 [1.13–3.37]), receiving government financial assistance (OR = 2.35 [1.16–4.76]), lower resilience (OR = 0.88 [0.85–0.93]), and higher loneliness (OR = 1.05 [1.02–1.08]) were significantly associated with psychiatric/psychological service use (detailed findings and sample characteristics are in Appendix pp 15–16). They also showed poorer overall functioning and health-related QoL, all *p* < 0.001 (Appendix pp 17).

#### Reasons for not seeking help among young people with MDE

Among those not receiving any psychiatric/psychological services, only 38.7% (n = 133) reported feeling a need to seek psychological counselling or medication. 127 of these young people (95.5%) provided further information regarding their reasons for not seeking help. Specifically, 57.5% (n = 73) rated “costs too much money” and 54.3% (n = 69) rated “talked to friends or relatives instead” as important reasons for not seeking help. The proportions of participants rating “important” or “very important” to other reasons for not seeking help were as follows: 44.1% (n = 56) “had problems with time, transportation, or scheduling”; 42.5% (n = 54) “wanted to handle the problem on your own”; 34.6% (n = 44) “not sure if available treatments were very effective”; 29.1% (n = 37) “unsure of where to go or who to see”, “too embarrassed”, and “afraid it might harm your school or professional career”; and 23.6% (n = 30) “worried that people would treat you differently”.

## Discussion

This study is among the first household-based epidemiological studies using diagnostic interviews to assess mental disorders amid periods of population stress, particularly in Asia. We explored trends in the prevalence of different major mental disorders in the youth population from 2019 to 2022, compared patterns of associated risk factors across periods, and explored rates of help-seeking across conditions. We found that around one to two in 10 young people in Hong Kong have a probable mental disorder or a major depressive episode during the past 12 months, which appear to be relatively high. Despite variations in the prevalence of MDE across periods, this rate remained persistently elevated even after the resolution of major population-level stressors. Further analyses revealed that MDE was significantly associated with poorer functioning and health-related quality of life. With these prevalence rates approaching those in the West,^7^, ^8^, ^9^ investment in cross-cultural and multi-site studies to investigate the phenomenon further would be needed to develop more timely and effective interventions for young people, particularly in view of the increasing population and global challenges in today’s world.

While influences of social unrest and the COVID-19 on mental health have already been reported in prior work,^2^, ^3^, ^4^^,^^12^ our study provided additional information concerning their additive influences on mental health in a representative epidemiological youth sample with consideration of a wide range of personal and environmental factors. By segregating our analyses into four periods, we showed that the prevalence of disorders and their risk factors could be subject to large-scale environmental changes. The changes in MDE prevalence indeed show resemblance to the trends in youth suicide rates in Japan,^37^ where a clear rise was seen during the peak COVID-19 period, followed by a gradual decline but which remained elevated compared to the pre-pandemic period. By further examining the factors associated with 12-month MDE across periods characterised by different societal stressors, we found that the three types of external stressors played different roles over time, with social unrest-related TEs being the only stressor type associated with MDE during period 1, followed by COVID-19 PEs during period 2, and personal SLEs during periods 3 and 4. These patterns of findings remained unchanged when different supplementary analyses were adopted, including the exclusion of other disorders as the outcome and multiple imputation.

Of note, despite the seemingly specific associations between stressor types and MDE across periods, the persistence of elevated disorder prevalence suggests that the impact of prior stressors would remain after their resolution (e.g., experiences of recent personal stressors being influenced by prior social unrest and COVID-19 events). Indeed, particularly for young people whose brains are still undergoing significant developments, the cumulative experiences of such stressors could have important long-lasting impacts (e.g., increased susceptibility to depression and other disorders).^38^^,^^39^ A number of local studies had in fact reported interactions between personal, social unrest-related, and pandemic-related stressors in their impact on depressive symptoms in young people.^18^, ^19^, ^20^ As in the study showing increased youth suicide rate in Japan during COVID-19,^37^ our previous work has also shown that both personal factors and COVID-19 stressors can contribute to suicidal ideation and attempts in the local youth population.^2^ The epidemiology of mental disorders thus requires consideration of the complex interactions between individual vulnerabilities, personal risk factors, and ongoing changes at the population level.^3^^,^^9^^,^^40^

Aside from environmental influences, individual factors including lower resilience, higher loneliness, frequent nightmares, and adverse childhood experiences showed consistent associations with MDE in both the younger and older youths, as well as across periods. In line with previous work,^9^^,^^23^^,^^41^ each of these factors may be taken as potential intervention targets, including during times of population stress. Meanwhile, poorer family relationships were associated with MDE only during period 1, which may be explained by differences in values and political stances among family members during the local protests. We also found fewer days of physical activity to be associated with MDE only during period 2 (early COVID-19 period) and smartphone overuse to be associated with MDE across the entire period and specifically during early periods of COVID-19. Complementing these observations, we had previously reported the negative impacts of restrictive pandemic measures on mental health and the protective effects of active leisure activities.^42^ It is also possible that these two factors could reinforce one another to further accentuate their impacts on mental health.^15^ A further examination of their interactions and implications not only during COVID-19 but also the post-pandemic era would be worthwhile.

Interestingly, it appears that social unrest-related TEs had a greater impact on the mental health of younger youths. One possible explanation might relate to the impact of smartphone overuse in this age group. The previous large family cohort study in Hong Kong has indeed found that spending more time on social media for sociopolitical news was related to elevated odds of probable depression and suspected PTSD.^4^ Particularly at a stage where the building of secure interpersonal relationships is crucial, the experience of peer and family conflicts—both online and offline—at the time of intense sociopolitical tension might have had a greater impact on the younger age group. Meanwhile, the reduced academic pressure due to school closure during the pandemic might also partially explain the lesser impact of COVID-19 stressors in the younger age group.^43^ In contrast, concerns about personal future and job insecurity due to COVID-19 could have been more prominent for older youths. While both the social unrest and the pandemic have now subsided, how young people would adjust to the post-crises lifestyle and future population-level threats would still require careful monitoring.

### Treatment gaps

Despite the impact of mental disorders on functioning and QoL, a large treatment gap remains–particularly for depression. We found that only 16.7% of those with 12-month MDE were using psychiatric/psychological services. This indicates only a slight increase from the last local epidemiological study in 2010–2013 (15.4%).^44^ Even when considering other services, the treatment gap we observed (73.4%) is still wider than those reported in previous studies (e.g., 35.5%–50.3% of those with any 12-month DSM-IV disorder in developed countries in the WHO World Mental Health Surveys^45^).

Notably, help-seeking was considerably lower for MDE compared to psychotic disorders (16.7% *vs* 61.5%). One reason might relate to the implementation of the city-wide specialised early intervention service (the Early Assessment Service for Young People with Psychosis [EASY]) since 2001, which has been successful in reducing burden on the system and duration of untreated psychosis.^46^ Similar to MDE, those with BD in our study also showed one of the lowest help-seeking rates (16.9%). Despite its prevalence being higher in our sample (2.3%) than those in the WMH surveys (0.4% for bipolar I and 0.3% for bipolar II^47^), this group of disorders has received markedly less attention in the literature and existing services. Expansion of services as the EASY to other conditions may help reduce these treatment gaps.

We acknowledge that some depressive symptoms experienced amid population-level stress could be transient. Longitudinal follow-up, especially in epidemiological samples, would be necessary for elucidating their trajectories, particularly as external stressors reside. Nevertheless, our data showed that help-seeking remains suboptimal even for past 30-day depressive episodes (27.2%), and only factors indicative of greater illness severity (e.g., poorer functioning) were predictive of help-seeking. Our further exploration revealed that perceiving services as too costly and preference for talking to friends and family remain to be the major reasons for not seeking professional help. While not captured in this study, it is possible that the fear of disclosure of personal information after the social unrest, as well as fear of COVID-19 infection, could have further prevented help-seeking.

With the increasing availability of youth-targeted mental health services free of cost in Hong Kong,^48^ these results highlight the need for further efforts in developing less stigmatising services and in improving their acceptability to reduce the treatment gap. Mainstreaming free mental health services, further investment in digital mental health screening and intervention, as well as improving public awareness of their availability, appear critical. Alongside the development and promotion of mental health services, improving mental health literacy among family members and peers would also be important as first-line support.

### Limitations and considerations

While our findings are relevant for other youth populations, specific contextual influences (e.g., differences in social distancing measures, perceptions of COVID-19) need to be accounted for. Potential differences in assessment mode (in-person/online) and motivation to participate in relation to external circumstances could also have implications on the findings. This will, nevertheless, inevitably require further consideration in future studies in view of the number of population and global changes today. Although the cross-period analyses enabled an exploration into changes in MDE prevalence over time using an epidemiological study design, we were unable to make inferences about within-person changes due to the cross-sectional nature of the study. Longitudinal investigations would help clarify the patterns of change in the individual manifestation of disorders and their trajectories.

In addition, while we considered the potential influence of migration on mental health, the design of the present study (i.e., invitations sent to addresses estimated with a local youth resident based on data from the 2016 Population By-Census) may not allow a more in-depth study into potential differences in the prevalence of mental disorders and their associated factors between locals and recent youth immigrants. With migration being an important risk factor for mental disorders,^49^^,^^50^ as well as the ongoing social changes in Hong Kong, this would be an area worthwhile for further studies.

We are aware that the health-related QoL values (using the EQ-5D-5L) were slightly higher in our sample compared to prior work.^51^ It is possible that the prolonged COVID-19 measures could have influenced these findings (e.g., reduced need to move about due to remote work/study, reduced social interaction opportunities). How contextual factors could influence measures of health-related QoL should be explored in the future.

Finally, we focused on five key conditions associated with the greatest role impairment in college students.^29^ Due to the relatively low prevalence of other conditions, the present study only examined specific factors associated with MDE. The lack of assessment of other conditions not included in this study could have resulted in an underestimation of overall mental disorder prevalence. Indeed, the examination of 12 mental health conditions in the Australian National Study of Mental Health^7^ might partially explain the higher prevalence of “any mental disorders” (39.6%) reported. Examining a wider range of conditions using similar diagnostic tools across populations in the future would be ideal.

### Conclusions

Our study provided a reference for future work in examining population mental health amid large-scale environmental changes. The importance of mitigating the impacts of not only personal but also contextual risks on youth mental health in future interventions and policies is highlighted. Continued efforts in identifying reliable and less stigmatising markers of psychiatric risks and designing specialised early intervention services for different conditions also remain a top priority.

## Contributors

SMYW and EYHC conceptualised the study presented in this manuscript and EYHC secured the funding. EYHC and CLMH were in charge of the project administration, YNS and CSMW managed the database, and EYHT, CHI, and WWKH recruited and interviewed participants. SMYW, EYHT, CHI, and WWKH analysed the data for this manuscript, and SMYW, EYHC, CLMH, YNS, EYHT, CHI, and WWKH have full access to all data of the study. SMYW did literature review, and both SMWY and EYHC drafted the initial versions of the manuscript. All authors contributed to the interpretation of study findings and revision of the manuscript, with additional comments provided by CLMH, YNS, PDM, CM, JVO, DM, PBJ, THL, and PCS. Further revisions were done by SWYW and EYHC. SMYW, EYHC, CLMH, YNS had final responsibility for the decision to submit for publication. All authors approved the final version of the manuscript.

## Data sharing statement

De-identified participant data in anonymised form will be available upon reasonable request and should be directed to the corresponding author.

## Editor note

The Lancet Group takes a neutral position with respect to territorial claims in published maps and institutional affiliations.

## Declaration of interests

EYHC has received speaker honoraria from Otsuka and research funding from Janssen. PJB is a trustee of the MQ Mental Health Research, Mental Health Research, UK, and Wolfson College, Cambridge, and has participated in the advisory board for MSD: The Future of Mental Health 2021.
