# Supplementary material for: Prevalence, time trends, and correlates of major depressive episode and other psychiatric conditions among young people amid major social unrest and COVID-19 in Hong Kong: a representative epidemiological study from 2019 to 2022
Source: Lancet Reg Health West Pac. 2023 Aug 19;40:100881. doi: 10.1016/j.lanwpc.2023.100881 (PMC10465929; doi:10.1016/j.lanwpc.2023.100881)
Supplement: Appendix [file mmc1.pdf]

Supplementary Material

Prevalence, time trends, and correlates of major depressive episode and other psychiatric conditions among young people amid major social unrest and COVID-19 in Hong Kong: a representative epidemiological study from 2019 to 2022

Index

Supplementary Material 1. Sample size calculation

Supplementary Material 2. Figures and Tables

Supplementary Figure S1. Rates of other 12-month DSM-IV conditions among young people without and with 12-month MDE in the HK-YES sample (n = 3340) 3

Supplementary Figure S2. Age differences in the prevalence of the five major DSM-IV conditions in the HK-YES sample (n = 3340) 4

Supplementary Table S1. Assessment of background, psychological factors, lifestyle characteristics, family functioning, and environmental stressors 5

Supplementary Table S2. Differences in characteristics between those without and with 12-month MDE in the epidemiological youth sample 6

Supplementary Table S3. Adjusted odds ratios for factors associated with 12-month MDE through the entire study period in the epidemiological youth sample 7

Supplementary Table S4. Adjusted odds ratios for factors associated with 12-month MDE through the entire study period in epidemiological youth sample after excluding those with other disorders 8

Supplementary Table S5. Age subgroup analysis showing adjusted odds ratios for factors associated with 12-month MDE within those aged 15–19 years and 20–25 years 9

Supplementary Table S6. Adjusted odds ratios for factors associated with 12-month MDE across periods amid ongoing population-level stressors, 2019–2022 (n = 3030) 10

Supplementary Table S7. Adjusted odds ratios for factors associated with 12-month MDE across periods amid ongoing population-level stressors after excluding those with other disorders, 2019–2022 11

Supplementary Table S8. Adjusted odds ratios for factors associated with 12-month MDE across periods amid ongoing population-level stressors, 2019–2022, in the imputed sample (n = 3340) 12

Supplementary Table S9. Functioning and health-related QoL associated with major DSM-IV psychiatric conditions in the epidemiological youth sample 13

Supplementary Table S10. Types of services currently used across major DSM-IV psychiatric conditions in epidemiological youth sample 14

Supplementary Table S11. Characteristics of participants with 12-month MDE who are and are not currently receiving psychiatric/psychological services in the epidemiological youth sample 15

Supplementary Table S12. Factors associated with current use of psychiatric/psychological services among young people with 12-month MDE in the epidemiological youth sample 16

Supplementary Table S13. Levels of functioning and quality of life among participants with 12-month MDE who are and are not currently receiving psychiatric/psychological services 17

Reference 18–19

Supplementary Material 1. Sample size calculation

The sample size of the HK-YES presented in the main text was derived using the following formula typically adopted in epidemiological and medical studies[1]:

$$n = \frac{Z^2 \, P(1-P)}{d^2} \times DEFF \,,$$

where *n* represents the sample size, *Z* represents the level of confidence (set at 95% = 1.96), *P* represents the expected prevalence of mental disorder, *d* represents the precision required corresponding to effect size, and DEFF represents design effect.

Since no youth-specific epidemiological study of mental health has been conducted in Hong Kong before the present study, we took reference to data from the last local epidemiological study of mental health of the general population (the HKMMS, 2010–2013[2]), specifically focusing on data from the youth subgroup of that study (i.e., those aged 16–25 years). The prevalence estimate of any depressive episode, generalised anxiety disorder (GAD), and mixed anxiety and depressive disorder was 10.7% in the HKMMS, as assessed using the Chinese Revised Clinical Interview Schedule (based on DSM and ICD diagnoses). Meanwhile, the prevalence of any common mental disorders (further considering other anxiety disorders, including phobias, panic disorder, and obsessive-compulsive disorder) was 11.3%. With an absolute precision of 1% with 95% confidence interval, the optimal sample size would be 3670 (if considering only depressive and anxiety episodes) and 3850 (if considering any common mental disorders). In the present HK-YES, we were able to obtain complete data on major mental disorders assessed using the CIDI-SC, including major depressive episode (MDE) and GAD, from 3340 young people.

Supplementary Material 2. Figures and Tables

Supplementary Figure S1. Rates of other 12-month DSM-IV conditions among young people without and with 12-month MDE in the HK-YES sample (n = 3340)

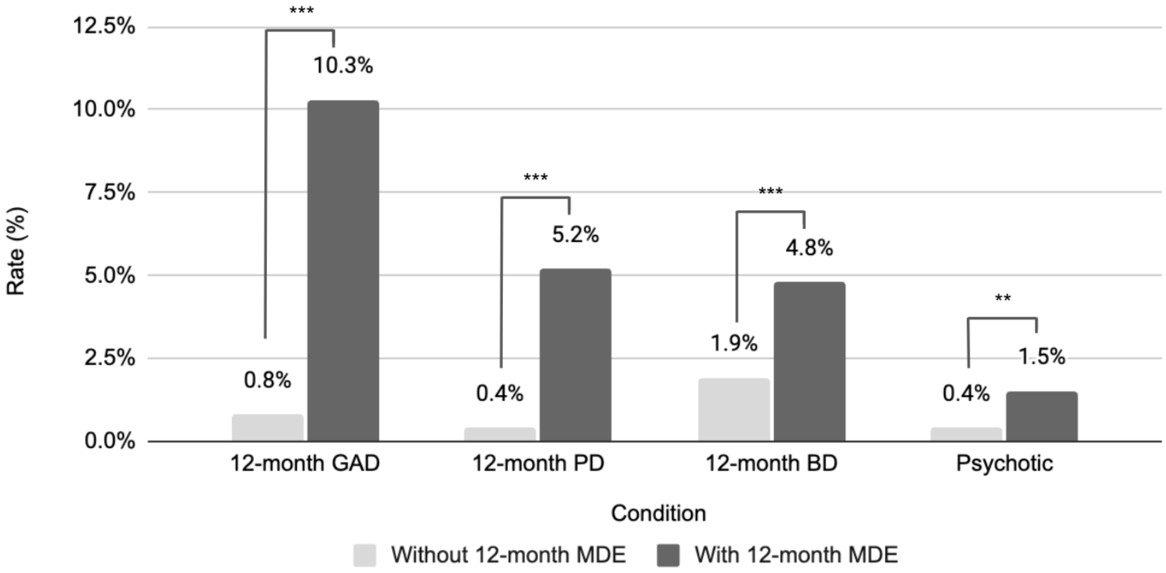

Note. Data are from 3340 participants of the entire HK-YES sample. MDE, GAD, PD, and BD were assessed using the interviewer-rated CIDI-SC according to the DSM-IV, while psychotic disorder was determined by experienced psychiatrists according to the Structured Clinical Interview for DSM. BD = bipolar disorder; GAD = generalized anxiety disorder; MDE = major depressive episode; PD = panic disorder. **p*<0.01, ***p*<0.001

Supplementary Figure S2. Age differences in the prevalence of the five major DSM-IV conditions in the HK-YES sample (n = 3340)

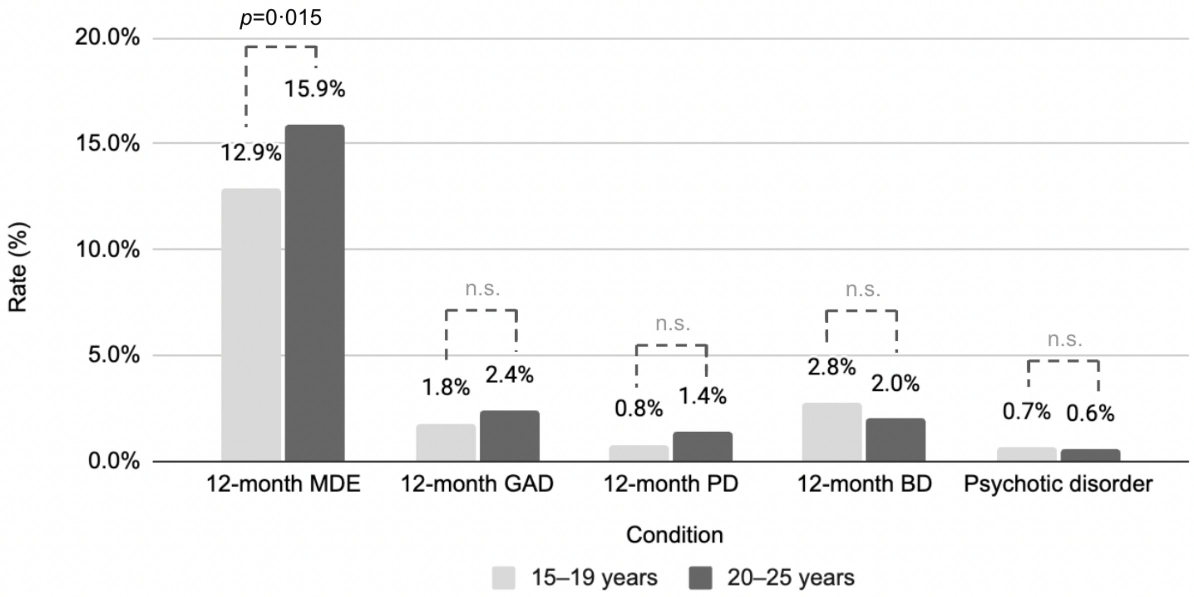

Note. Data are from 3340 participants of the entire HK-YES sample. Prevalence data are unweighted in this analysis. MDE, GAD, PD, and BD were assessed using the interviewer-rated CIDI-SC according to the DSM-IV, while psychotic disorder was determined by experienced psychiatrists according to the Structured Clinical Interview for DSM. BD = bipolar disorder; GAD = generalized anxiety disorder; MDE = major depressive episode; PD = panic disorder.

Supplementary Table S1. Assessment of background, psychological factors, lifestyle characteristics, family functioning, and environmental stressors

Variable	Measure
**Background factors**	● **Government financial assistance**: Participants were asked whether his/her family is currently receiving any form of government financial assistance (e.g., disability allowance, medical fee waiver, emergency relief fund, traffic accident victims assistance scheme). This offers an indicator of the participant's socioeconomic status as in previous studies, which may be less challenging for young people in reporting compared to parental or household income and parental education level.[1–4]<br>● **Childhood adversity**: Items pertaining to the experience of childhood adversity prior to the age of 17 were selected from the CIDI-3.0[5] childhood section for the current study. Four types of adversity were assessed, including emotional abuse (three items assessing the frequency with the participant had been insulted, said hurtful things, or emotionally abused by the caregiver), physical abuse (two items examining the frequency with which the participant had been pushed, hit, or bruised the caregiver), neglect (two items assessing the frequency of having inadequate supervision and having to do dangerous, age-inappropriate tasks), and sexual abuse (two items assessing experiences of sexual assault, unwanted sexual contact or abuse). Each item was rated on a 5-point Likert scale (0 "never" to 4 "very often"). A score of 3 or above on any of the items was defined as having experienced childhood adversity.
**Psychological factors**	● **Resilience**: 10-item Connor-Davidson Resilience Scale (CD-RISC-10)[6,7] (10 items, score range: 0–40). The psychometric properties and normative data of the measure in Hong Kong adolescents have been reported.[8] (Cronbach's alpha: α=0.90)<br>● **Loneliness**: UCLA Loneliness Scale full version (UCLA-LS)[9] (20 items, score range: 20–80). The UCLA-LS measure is among the most widely adopted measure of loneliness in adolescents and has been validated in Chinese.[10,11] (Cronbach's alpha: α=0.65)
**Digitalisation and lifestyle**	● **Smartphone overuse**: An adapted version of the 26-item Revised Chen Internet Addiction Scale (CIAS-R),[12,13] which captures five key domains of behavioral addictions associated with the use of the Internet through smartphones (compulsive use, tolerance, withdrawal, problems with interpersonal relationships, and time management). Each item was rated on a 4-point Likert scale and summed to compute a composite score (score range: 26–104). A score of 67 or above indicates probable smartphone overuse.[14] This adapted version of the CIAS-R has been presented in our prior work examining the impact of smartphone overuse on 1-year severe depressive symptoms.[15] (Cronbach's alpha: α=0.92)<br>● **Frequent nightmares**: An item from the Pittsburgh Sleep Quality Index (PSQI)[16,17] was used, which assesses the frequency of "having bad dreams" (which translates directly to "nightmares" in Chinese) during the past month. The item was rated on a 4-point scale: (i) no nightmare, (ii) <1 time a week, (iii) 1–2 times a week, and (iv) ≥3 times a week. As in previous studies,[18–20] frequent nightmares were defined as having ≥1 nightmare a week during the past month. We have also used this measure in our prior work and found support for its role in 1-year moderate-to-severe depressive symptoms, as well as MDE.[21]<br>● **Physical activity**: Two items from the International Physical Activity Questionnaire (IPAQ),[22,23] which asks participants the number of days during the past 7 days they had engaged in moderate-intensity and vigorous-intensity physical activity, respectively. Responses to these two items were averaged to define days of moderate-to-vigorous level physical activity in this study as in our prior work.[21]
**Family functioning**	● **Family dysfunction**: Brief Family Relationship Scale (BFRS)[24] (16 items, score range: 0–48). A higher score denotes poorer family functioning. A higher score denotes poorer family functioning. The measure has been validated in adolescents.[24] (Cronbach's alpha: α=0.90)
**Personal and population-level stressors**	● **Personal stressful life events (SLEs)**: The List of Threatening Events (LTE),[25] which consists of 12 types of personal life stressors in a checklist format (e.g., death of a first-degree or second-degree relative, major financial crisis). In view of its use in the epidemiologic youth sample, the options "sacked from job" and "unemployment" were provided with the alternatives "expelled from school" and "dropped out of school", respectively. An "others" option was also included, resulting in a total of 13 SLEs possible. The use of this measure has also been reported in our prior work.[21]<br>● **Social unrest-related TEs**: Three key experiences of TEs since June 2019 (the beginning of the series of social unrest in Hong Kong) were assessed using a checklist format, including "crowd dispersal by the use of force", "arrest or detention", and "media viewing of others being physically attacked". These experiences were designed to capture the more prominent experiences of the social unrest at the time based on prior work.[26–28]<br>● **COVID-19 pandemic-related events (PEs)**: A checklist capturing four key experiences of COVID-19 PEs since its local outbreak, including "having sufficient gears" (reversed coded), "increased personal and rest time due to remote work/school" (reversed coded), "increased conflicts with family due to remote work/school", and "increased work/studies hours due to remote work/school". Each item was rated on a 5-point Likert scale ("completely disagree" to "completely agree") before November 2020 and in the form of a checklist thereafter. A rating of "completely disagree" or "disagree" for the items "having sufficient gears" and "increased personal and rest time" and a rating of "agree" or "completely agree" for the other two items were recoded into 1 for those with their assessments completed before November 2020. A score of 0 was given to those who completed their assessments before the local outbreak of COVID-19. The use of this measure has also been reported in our prior work.[21,28]

Supplementary Table S2. Differences in characteristics between those without and with 12-month MDE in the epidemiological youth sample

Variables	Factors associated with MDE	
	No 12-month MDE (n = 2594)	Has 12-month MDE (n = 436)
**Background factors**		
Female sex, n (%)	**1450 (55·9%)**	**313 (71·8%)**
Age	19·81 (2·81)	20·10 (2·64)
Born in Hong Kong, n (%)	2087 (80·5%)	350 (80·3%)
Receiving government financial assistance, n (%)[a]	243 (9·4%)	44 (10·1%)
Has childhood adversity, n (%)	**820 (31·6%)**	**255 (58·5%)**
**Psychological factors**		
Resilience (CD-RISC-10)	**24·68 (5·98)**	**20·17 (6·62)**
Loneliness (UCLA-LS)	**42·94 (8·60)**	**49·70 (9·20)**
**Digitalisation and lifestyle**		
Smartphone overuse (CIAS-R ≥ 67), n (%)	**709 (27·3%)**	**196 (45·0%)**
Frequent nightmares (≥1 / week), n (%)	**354 (13·6%)**	**154 (35·3%)**
Days of regular physical activity (past week)	**1·74 (1·57)**	**1·50 (1·45)**
**Family functioning**		
Family dysfunction (BFRS)	**19·07 (6·77)**	**23·30 (8·19)**
**Perceived stress and stressors**		
≥2 personal SLEs, n (%)	**527 (20·3%)**	**147 (33·7%)**
≥2 social unrest-related TEs, n (%)	**469 (18·1%)**	**107 (24·5%)**
≥2 COVID-19 stressors, n (%)[b]	**870 (35·3%)**	**182 (44·9%)**

[a]Data on any government financial assistance received were available from 3024 participants.
[b]Data on COVID-19 stressors were collected since March 2020 and were available from 2870 participants.
Note· Data are from 3030 participants of the HK-YES· Values are presented in the form of mean (SD), unless otherwise stated· Statistics significant after accounting for multiple comparisons (0·05/21=0·0024) in the univariate analyses are boldfaced. BFI = Big Five Inventory; BFRS = Brief Family Relationship Scale; CD-RISC-10 = Connor-Davidson Resilience Scale 10-Item; CIAS-R = Revised Chen Internet Addiction Scale; MDE = major depressive episode; PSQI = Pittsburgh Sleep Quality Index; SLEs = Stressful Life Events; UCLA-LS = 20-item UCLA Loneliness Scale.

Supplementary Table S3. Adjusted odds ratios for factors associated with 12-month MDE through the entire study period in the epidemiological youth sample

Variables	12-month MDE (n = 436)	
	Adjusted OR (95% CI)	*p*
**Background factors**		
Male sex	1 [Ref]	
Female sex	**1·86 (1·44–2·40)**	**<0·001**
Age	1·02 (0·97–1·06)	0·42
No childhood adversity	1 [Ref]	
Has childhood adversity	**1·92 (1·50–2·48)**	**<0·001**
**Psychological factors**		
Resilience (CD-RISC-10)	**0·94 (0·92–0·96)**	**<0·001**
Loneliness (UCLA-LS)	**1·06 (1·05–1·08)**	**<0·001**
**Digitalisation and lifestyle**		
No smartphone overuse (CIAS-R < 67)	1 [Ref]	
Smartphone overuse (CIAS-R ≥ 67)	**1·27 (1·00–1·62)**	**0·050**
No frequent nightmares (<1 / week)	1 [Ref]	
Frequent nightmares (≥1 / week)	**2·25 (1·73–2·93)**	**<0·001**
Days of regular exercise (past week)	0·94 (0·87–1·02)	0·15
**Family relationship**		
Family dysfunction (BFRS)	1·02 (1·00–1·04)	0·081
**Personal and population-level stressors**		
<2 personal SLEs	1 [Ref]	
≥2 personal SLEs	**1·59 (1·23–2·05)**	**<0·001**
<2 social unrest-related TEs	1 [Ref]	
≥2 social unrest-related TEs	**1·44 (1·09–1·92)**	**0·011**
<2 COVID-19 stressors[b]	1 [Ref]	
≥2 COVID-19 stressors[b]	**1·33 (1·05–1·69)**	**0·019**

[a]Data on any government financial assistance received were available from 3024 participants.

[b]Data on COVID-19 stressors were collected since March 2020 and were available from 2870 participants.

Note. Data are from 3030 participants of the HK-YES. All variables significant in the univariate analyses after accounting for multiple comparisons were adjusted for in these multivariable logistic regression models. Values significant at the $p < 0.05$ level are boldfaced. BFRS = Brief Family Relationship Scale; CD-RISC-10 = Connor-Davidson Resilience Scale 10-Item; CIAS-R = Revised Chen Internet Addiction Scale; MDE = major depressive episode; PEs = COVID-19 pandemic-related events; PSQI = Pittsburgh Sleep Quality Index; SLEs = Stressful Life Events; TEs = social unrest-related traumatic events; UCLA-LS = 20-item UCLA Loneliness Scale.

Supplementary Table S4. Adjusted odds ratios for factors associated with 12-month MDE through the entire study period in epidemiological youth sample after excluding those with other disorders

Variable	12-month MDE during the entire study period (n = 436)	
	Adjusted OR (95% CI)	*p*
**Background factors**		
Male sex	1 [Ref]	
Female sex	**1·84 (1·42–2·39)**	**<0·001**
Age	1·02 (0·98–1·07)	0·37
No childhood adversity	1 [Ref]	
Has childhood adversity	**2·04 (1·58–2·63)**	**<0·001**
**Psychological factors**		
Resilience (CD-RISC-10)	**0·94 (0·92–0·96)**	**<0·001**
Loneliness (UCLA-LS)	**1·06 (1·05–1·08)**	**<0·001**
**Lifestyle factors**		
No smartphone overuse (CIAS-R < 67)	1 [Ref]	
Smartphone overuse (CIAS-R ≥ 67)	**1·31 (1·02–1·67)**	**0·031**
No frequent nightmares (<1 / week)	1 [Ref]	
Frequent nightmares (≥1 / week)	**2·31 (1·77–3·01)**	**<0·001**
Days of regular physical activity (past week)	0·96 (0·89–1·04)	0·34
**Family functioning**		
Poor family functioning (BFRS)	1·02 (1·00–1·03)	0·10
**Personal and population-level stressors**		
<2 personal SLEs	1 [Ref]	
≥2 personal SLEs	**1·66 (1·28–2·15)**	**<0·001**
<2 social unrest-related TEs	1 [Ref]	
≥2 social unrest-related TEs	**1·50 (1·12–1·99)**	**0·006**
<2 COVID-19 PEs	1 [Ref]	
≥2 COVID-19 PEs	**1·33 (1·04–1·68)**	**0·021**

Note. Data are from 2947 participants among the n = 3030 HK-YES sample after excluding those with other disorders. Statistics significant at the p<0.05 level are boldfaced. BFRS = Brief Family Relationship Scale; CD-RISC-10 = Connor-Davidson Resilience Scale 10-Item; CIAS-R = Revised Chen Internet Addiction Scale; MDE = major depressive episode; PEs = COVID-19 pandemic-related events; SLEs = Stressful Life Events; TEs = social unrest-related traumatic events; UCLA-LS = 20-item UCLA Loneliness Scale.

Supplementary Table S5. Age subgroup analysis showing adjusted odds ratios for factors associated with 12-month MDE within those aged 15–19 years and 20–25 years

Variables	Aged 15–19 years (n = 1368)		Aged 20–25 years (n = 1662)	
	12-month MDE (n = 176)		12-month MDE (n = 260)	
	Adjusted OR (95% CI)	*p*	Adjusted OR (95% CI)	*p*
**Background factors**				
Male sex	1 [Ref]		1 [Ref]	
Female sex	**2·07 (1·38–3·11)**	**<0·001**	**1·78 (1·28–2·49)**	**0·001**
No childhood adversity	1 [Ref]		1 [Ref]	
Has childhood adversity	**2·27 (1·57–3·29)**	**<0·001**	**1·98 (1·45–2·70)**	**<0·001**
Not receiving government financial assistance	··	··	··	··
Receiving government financial assistance[a]	··	··	··	··
Born in Hong Kong	··	··	··	··
Not born in Hong Kong	··	··	··	··
**Psychological factors**				
Resilience (CD-RISC-10)	**0·94 (0·91–0·97)**	**<0·001**	**0·93 (0·91–0·96)**	**<0·001**
Loneliness (UCLA-LS)	**1·07 (1·04–1·09)**	**<0·001**	**1·07 (1·05–1·09)**	**<0·001**
**Digitalisation and lifestyle**				
No smartphone overuse (CIAS-R < 67)	1 [Ref]		··	··
Smartphone overuse (CIAS-R ≥ 67)	**1·63 (1·12–2·37)**	**0·011**	··	··
No frequent nightmares (<1 / week)	1 [Ref]		1 [Ref]	
Frequent nightmares (≥1 / week)	**2·87 (1·91–4·32)**	**<0·001**	**1·96 (1·39–2·76)**	**<0·001**
Days of regular exercise (past week)	··	··	**0·89 (0·80–0·99)**	**0·034**
**Family relationship**				
Family dysfunction (BFRS)	··	··	··	··
**Personal and population-level stressors**				
<2 personal SLEs	1 [Ref]		1 [Ref]	
≥2 personal SLEs	**1·58 (1·05–2·38)**	**0·028**	**1·66 (1·19–2·31)**	**0·003**
<2 social unrest-related TEs	1 [Ref]		1 [Ref]	
≥2 social unrest-related TEs	**1·73 (1·06–2·80)**	**0·002**	1·37 (0·97–1·94)	0·074
<2 COVID-19 stressors[b]	··	··	1 [Ref]	
≥2 COVID-19 stressors[b]	··	··	**1·53 (1·12–2·09)**	**0·007**

[a]Data on any government financial assistance received were available from 1366 and 1658 participants in the 15–19 and 20–25 years subgroups, respectively. .

[b]Data on COVID-19 stressors were collected since March 2020 and were available from 1308 and 1562 participants in the 15–19 and 20–25 years subgroups, respectively.

Note. Data are from 3030 participants of the HK-YES. The backward selection method was used for the multivariable logistic regression model. BFRS = Brief Family Relationship Scale; CD-RISC-10 = Connor-Davidson Resilience Scale 10-Item; CIAS-R = Revised Chen Internet Addiction Scale; MDE = major depressive episode; PEs = COVID-19 pandemic-related events; PSQI = Pittsburgh Sleep Quality Index; SLEs = Stressful Life Events; TEs = social unrest-related traumatic events; UCLA-LS = 20-item UCLA Loneliness Scale.

Supplementary Table S6. Adjusted odds ratios for factors associated with 12-month MDE across periods amid ongoing population-level stressors, 2019–2022 (n = 3030)

	12-month MDE			
	Period 1 (May 2019-Jun 2020) (n = 100 / 739)	Period 2 (Jul 2020-Dec 2020) (n = 126 / 706)	Period 3 (Jan 2021-Jun 2021) (n = 112 / 851)	Period 4 (Jul 2021-Jun 2022) (n = 98 / 734)
Variable	Adjusted OR (95% CI)	Adjusted OR (95% CI)	Adjusted OR (95% CI)	Adjusted OR (95% CI)
Background factors				
Male sex	1 [Ref]	1 [Ref]	1 [Ref]	1 [Ref]
Female sex	**2·07 (1·20–3·59)****	**2·08 (1·29–3·36)****	**2·20 (1·32–3·69)****	1·49 (0·89–2·48)
Age	0·91 (0·83–1·00)	1·05 (0·97–1·14)	1·08 (0·99–1·18)	1·02 (0·93–1·11)
No childhood adversity	1 [Ref]	1 [Ref]	1 [Ref]	1 [Ref]
Has childhood adversity	**2·07 (1·24–3·45)****	**1·69 (1·05–2·73)***	**1·64 (1·01–2·65)***	**2·07 (1·21–3·54)****
Psychological factors				
Resilience (CD-RISC-10)	**0·92 (0·89–0·96)*****	**0·96 (0·92–1·00)***	**0·90 (0·87–0·94)*****	**0·94 (0·90–0·98)****
Loneliness (UCLA-LS)	**1·07 (1·03–1·10)*****	**1·04 (1·01–1·07)****	**1·06 (1·03–1·09)*****	**1·08 (1·05–1·11)*****
Lifestyle factors				
No smartphone overuse (CIAS-R < 67)	1 [Ref]	1 [Ref]	1 [Ref]	1 [Ref]
Smartphone overuse (CIAS-R ≥ 67)	0·98 (0·60–1·62)	**1·76 (1·12–2·76)***	1·20 (0·75–1·93)	1·40 (0·85–2·32)
No frequent nightmares (<1 / week)	1 [Ref]	1 [Ref]	1 [Ref]	1 [Ref]
Frequent nightmares (≥1 / week)	**2·39 (1·44–3·98)****	**2·48 (1·50–4·09)*****	**2·23 (1·29–3·88)****	**2·14 (1·24–3·71)****
Days of regular physical activity (past week)	1·02 (0·87–1·19)	**0·85 (0·72–0·99)***	0·99 (0·85–1·16)	1·02 (0·86–1·20)
Family functioning				
Poor family functioning (BFRS)	**1·04 (1·01–1·08)***	1·02 (0·99–1·06)	0·99 (0·96–1·03)	1·02 (0·98–1·06)
Personal and population-level stressors				
<2 personal SLEs	1 [Ref]	1 [Ref]	1 [Ref]	1 [Ref]
≥2 personal SLEs	1·00 (0·59–1·69)	1·37 (0·86–2·18)	**2·15 (1·28–3·62)****	**1·93 (1·12–3·33)***
<2 social unrest-related TEs	1 [Ref]	1 [Ref]	1 [Ref]	1 [Ref]
≥2 social unrest-related TEs	**2·04 (1·23–3·39)****	1·38 (0·83–2·30)	1·41 (0·71–2·80)	0·93 (0·47–1·83)
<2 COVID-19 PEs	1 [Ref]	1 [Ref]	1 [Ref]	1 [Ref]
≥2 COVID-19 PEs	··	**1·83 (1·15–2·90)***	1·39 (0·87–2·22)	1·00 (0·61–1·63)

Note. All variables significant in the univariate analyses after accounting for multiple comparisons were adjusted for in these multivariable logistic regression models (sex, age, and childhood adversity were included in all models regardless of their significance). Statistics significant at the $p<0.05$ level are boldfaced. BFRS = Brief Family Relationship Scale; CD-RISC-10 = Connor-Davidson Resilience Scale 10-Item; CIAS-R = Revised Chen Internet Addiction Scale; MDE = major depressive episode; PEs = COVID-19 pandemic-related events; SLEs = Stressful Life Events; TEs = social unrest-related traumatic events; UCLA-LS = 20-item UCLA Loneliness Scale.
*$p < 0.05$, **$p < 0.01$, ***$p < 0.001$

Supplementary Table S7. Adjusted odds ratios for factors associated with 12-month MDE across periods amid ongoing population-level stressors after excluding those with other disorders, 2019–2022

Variable	12-month MDE			
	Period 1 (May 2019-Jun 2020) (n = 100 / 718)	Period 2 (Jul 2020-Dec 2020) (n = 126 / 684)	Period 3 (Jan 2021-Jun 2021) (n = 112 / 830)	Period 4 (Jul 2021-Jun 2022) (n = 98 / 715)
	Adjusted OR (95% CI)	Adjusted OR (95% CI)	Adjusted OR (95% CI)	Adjusted OR (95% CI)
Background factors				
Male sex	1 [Ref]	1 [Ref]	1 [Ref]	1 [Ref]
Female sex	**2·06 (1·18–3·58)****	**2·05 (1·26–3·32)****	**2·21 (1·32–3·72)****	1·50 (0·90–2·51)
Age	**0·91 (0·83–1·00)***	1·05 (0·97–1·15)	1·08 (0·99–1·18)	1·02 (0·93–1·12)
No childhood adversity	1 [Ref]	1 [Ref]	1 [Ref]	1 [Ref]
Has childhood adversity	**2·26 (1·34–3·80)****	**1·77 (1·09–2·87)***	**1·79 (1·10–2·91)***	**2·12 (1·23–3·65)****
Psychological factors				
Resilience (CD-RISC-10)	**0·91 (0·87–0·95)*****	**0·96 (0·92–1·00)***	**0·90 (0·86–0·94)*****	**0·94 (0·90–0·98)****
Loneliness (UCLA-LS)	**1·07 (1·03–1·10)*****	**1·04 (1·01–1·07)****	**1·06 (1·03–1·09)*****	**1·08 (1·05–1·12)*****
Lifestyle factors				
No smartphone overuse (CIAS-R < 67)	1 [Ref]	1 [Ref]	1 [Ref]	1 [Ref]
Smartphone overuse (CIAS-R ≥ 67)	1·00 (0·60–1·66)	**1·81 (1·15–2·86)***	1·24 (0·77–1·99)	1·49 (0·89–2·48)
No frequent nightmares (<1 / week)	1 [Ref]	1 [Ref]	1 [Ref]	1 [Ref]
Frequent nightmares (≥1 / week)	**2·42 (1·45–4·04)****	**2·51 (1·51–4·17)*****	**2·29 (1·31–4·01)****	**2·30 (1·32–4·02)****
Days of regular physical activity (past week)	1·04 (0·89–1·23)	**0·85 (0·72–0·99)***	1·01 (0·86–1·18)	1·04 (0·89–1·23)
Family functioning				
Poor family functioning (BFRS)	**1·04 (1·01–1·08)***	1·02 (0·98–1·05)	0·99 (0·96–1·03)	1·02 (0·98–1·06)
Personal and population-level stressors				
<2 personal SLEs	1 [Ref]	1 [Ref]	1 [Ref]	1 [Ref]
≥2 personal SLEs	1·04 (0·61–1·76)	1·39 (0·87–2·23)	**2·33 (1·37–3·95)****	**2·02 (1·17–3·51)***
<2 social unrest-related TEs	1 [Ref]	1 [Ref]	1 [Ref]	1 [Ref]
≥2 social unrest-related TEs	**2·27 (1·36–3·81)****	1·46 (0·88–2·43)	1·38 (0·69–2·77)	0·97 (0·49–1·92)
<2 COVID-19 PEs	1 [Ref]	1 [Ref]	1 [Ref]	1 [Ref]
≥2 COVID-19 PEs	–	**1·74 (1·09–2·77)***	1·39 (0·86–2·23)	1·02 (0·62–1·68)

Note. Data are from 2947 participants among the n = 3030 HK-YES sample after excluding those with other disorders. Statistics significant at the p<0.05 level are boldfaced. BFRS = Brief Family Relationship Scale; CD-RISC-10 = Connor-Davidson Resilience Scale 10-Item; CIAS-R = Revised Chen Internet Addiction Scale; MDE = major depressive episode; PEs = COVID-19 pandemic-related events; SLEs = Stressful Life Events; TEs = social unrest-related traumatic events; UCLA-LS = 20-item UCLA Loneliness Scale. *p < 0.05, **p < 0.01, ***p < 0.001

Supplementary Table S8. Adjusted odds ratios for factors associated with 12-month MDE across periods amid ongoing population-level stressors, 2019–2022, in the imputed sample (n = 3340)

Variable	12-month MDE			
	Period 1 (May 2019-Jun 2020) (n = 111 / 839)	Period 2 (Jul 2020-Dec 2020) (n = 138 / 764)	Period 3 (Jan 2021-Jun 2021) (n = 128 / 912)	Period 4 (Jul 2021-Jun 2022) (n = 109 / 825)
	Adjusted OR (95% CI)	Adjusted OR (95% CI)	Adjusted OR (95% CI)	Adjusted OR (95% CI)
Background factors				
Male sex	1 [Ref]	1 [Ref]	1 [Ref]	1 [Ref]
Female sex	**1·98 (1·20–3·28)****	**1·84 (1·18–2·86)****	**1·98 (1·23–3·18)****	1·19 (0·74–1·91)
Age	0·94 (0·86–1·03)	1·04 (0·96–1·12)	1·07 (0·99–1·16)	1·00 (0·93–1·09)
No childhood adversity	1 [Ref]	1 [Ref]	1 [Ref]	1 [Ref]
Has childhood adversity	**1·92 (1·18–3·12)****	**1·62 (1·03–2·53)***	**2·12 (1·35–3·33)***	**2·30 (1·40–3·80)****
Psychological factors				
Resilience (CD-RISC-10)	**0·92 (0·88–0·96)*****	0·97 (0·93–1·00)	**0·91 (0·88–0·95)*****	**0·93 (0·89–0·96)*****
Loneliness (UCLA-LS)	**1·07 (1·03–1·10)*****	**1·04 (1·01–1·07)****	**1·06 (1·02–1·09)*****	**1·06 (1·03–1·09)*****
Lifestyle factors				
No smartphone overuse (CIAS-R < 67)	1 [Ref]	1 [Ref]	1 [Ref]	1 [Ref]
Smartphone overuse (CIAS-R ≥ 67)	0·91 (0·56–1·46)	**1·75 (1·15–2·67)***	1·25 (0·81–1·95)	1·55 (0·97–2·49)
No frequent nightmares (<1 / week)	1 [Ref]	1 [Ref]	1 [Ref]	1 [Ref]
Frequent nightmares (≥1 / week)	**2·00 (1·23–3·24)****	**2·46 (1·54–3·92)*****	**2·49 (1·50–4·13)*****	**2·03 (1·21–3·42)****
Days of regular physical activity (past week)	1·01 (0·87–1·18)	0·87 (0·75–1·01)	1·00 (0·86–1·16)	0·98 (0·84–1·15)
Family functioning				
Poor family functioning (BFRS)	1·03 (1·00–1·07)	1·03 (1·00–1·06)	1·00 (0·97–1·03)	1·01 (0·98–1·05)
Personal and population-level stressors				
<2 personal SLEs	1 [Ref]	1 [Ref]	1 [Ref]	1 [Ref]
≥2 personal SLEs	1·05 (0·64–1·72)	1·27 (0·82–1·97)	**2·12 (1·30–3·45)****	**1·73 (1·03–2·90)***
<2 social unrest-related TEs	1 [Ref]	1 [Ref]	1 [Ref]	1 [Ref]
≥2 social unrest-related TEs	**2·09 (1·29–3·39)****	1·30 (0·80–2·11)	1·18 (0·60–2·33)	1·01 (0·53–1·93)
<2 COVID-19 PEs	1 [Ref]	1 [Ref]	1 [Ref]	1 [Ref]
≥2 COVID-19 PEs	–	**1·59 (1·03–2·47)***	1·14 (0·74–1·75)	0·97 (0·61–1·55)

Note. Data are based on the multiple imputation models, with 3340 participants from the HK-YES. Statistics significant at the $p<0.05$ level are boldfaced. BFRS = Brief Family Relationship Scale; CD-RISC-10 = Connor-Davidson Resilience Scale 10-Item; CIAS-R = Revised Chen Internet Addiction Scale; MDE = major depressive episode; PEs = COVID-19 pandemic-related events; SLEs = Stressful Life Events; TEs = social unrest-related traumatic events; UCLA-LS = 20-item UCLA Loneliness Scale. **p* < 0.05, ***p* < 0.01, ****p* < 0.001

Supplementary Table S9. Functioning and health-related QoL associated with major DSM-IV psychiatric conditions in the epidemiological youth sample

Variables	Sample (n = 3030)	No 12-month MDE (n = 2594)	Has 12-month MDE (n = 436)	*p*
**Functioning**				
Social and occupational functioning (SOFAS)	82·67 (7·71)	**83·60 (7·19)**	**77·09 (8·35)**	**<0·001**
Days of reduced productivity	2·19 (4·69)	**1·63 (3·80)**	**5·53 (7·35)**	**<0·001**
Days of lost productivity	0·40 (2·09)	**0·19 (1·31)**	**1·64 (4·29)**	**<0·001**
**Health-related quality of life (QoL)**				
Overall health-related QoL (EQ-5D-5L)	0·95 (0·08)	**0·96 (0·07)**	**0·90 (0·11)**	**<0·001**
Physical health QoL (PCS-12)	52·48 (6·99)	**52·84 (6·69)**	**50·38 (8·24)**	**<0·001**
Mental health QoL (MCS-12)	41·38 (11·51)	**43·10 (10·54)**	**31·17 (11·77)**	**<0·001**
Global mental well-being (WHO-5)	54·49 (18·99)	**56·82 (18·15)**	**40·64 (17·97)**	**<0·001**

Note. Data are from 3030 participants of the HK-YES. All values are presented in the form of mean (SD). EQ-5D-5L = EuroQol-5D; MDE = major depressive episode; MCS-12 = Mental Component of the 12-Item Short Form Survey (SF-12); PCS-12 = Physical Component of the SF-12; SOFAS = Social and Occupational Functioning Assessment Scale; WHO-5 = The World Health Organisation–Five Well-Being Index.

Supplementary Table S10. Types of services currently used across major DSM-IV psychiatric conditions in epidemiological youth sample

	12-month MDE (n = 436)	30-day MDE (n = 92)	12-month GAD (n = 64)	30-day GAD (n = 50)	12-month PD (n = 32)	12-month BD (n = 71)	Psychotic disorder (n = 13)
**Type of service used**							
Psychiatric/Psychological services	73 (16·7%)	25 (27·2%)	18 (28·1%)	17 (34·0%)	14 (43·8%)	12 (16·9%)	8 (61·5%)
Other services only[a]	43 (9·9%)	9 (9·8%)	6 (9·4%)	5 (10·0%)	1 (3·1%)	5 (7·0%)	1 (7·7%)
No	320 (73·4%)	58 (63·0%)	40 (62·5%)	28 (56·0%)	17 (53·1%)	54 (76·1%)	4 (30·8%)
**Service providers**							
Psychiatrist	70 (16·1%)	24 (26·1%)	18 (28·1%)	17 (34·0%)	13 (40·6%)	12 (16·9%)	8 (61·5%)
Psychologist	30 (6·9%)	12 (13·0%)	7 (10·9%)	6 (12·0%)	8 (25·0%)	7 (9·9%)	2 (15·4%)
Community psychiatric nurse	0 (0·0%)	0 (0·0%)	0 (0·0%)	0 (0·0%)	0 (0·0%)	0 (0·0%)	0 (0·0%)
General practitioner (private)	4 (0·9%)	0 (0·0%)	0 (0·0%)	0 (0·0%)	0 (0·0%)	0 (0·0%)	0 (0·0%)
Non-psychiatric clinical / nursing services	4 (0·9%)	1 (1·1%)	1 (1·6%)	1 (2·0%)	1 (3·1%)	0 (0·0%)	0 (0·0%)
Social worker (public)	56 (12·8%)	16 (17·4%)	9 (14·1%)	7 (14·0%)	7 (21·9%)	12 (16·9%)	4 (30·8%)
Occupational therapist	5 (1·1%)	2 (2·2%)	2 (3·1%)	2 (4·0%)	1 (3·1%)	1 (1·4%)	1 (7·7%)
Traditional Chinese practitioner	4 (0·9%)	1 (1·1%)	1 (1·6%)	1 (2·0%)	0 (0·0%)	0 (0·0%)	0 (0·0%)
Religious services	2 (0·5%)	0 (0·0%)	0 (0·0%)	0 (0·0%)	0 (0·0%)	0 (0·0%)	0 (0·0%)
Day center	0 (0·0%)	0 (0·0%)	0 (0·0%)	0 (0·0%)	0 (0·0%)	0 (0·0%)	0 (0·0%)
Other	15 (3·4%)	5 (5·4%)	2 (3·1%)	2 (4·0%)	2 (6·3%)	2 (2·8%)	2 (15·4%)

[a] Other services include those from general practitioners, non-psychiatric clinical / nursing services, social workers, occupational therapists, traditional Chinese practitioners, religious services, day center, and others.

Note. Data are from 3030 participants of the HK-YES. MDE, GAD, PD, and BD were assessed using the interviewer-rated CIDI-SC according to the DSM-IV. Psychotic disorder was confirmed by experienced psychiatrists according to the Structured Clinical Interview for DSM. BD = bipolar disorder; GAD = generalized anxiety disorder; MDE = major depressive episode; PD = panic disorder.

Supplementary Table S11. Characteristics of participants with 12-month MDE who are and are not currently receiving psychiatric/psychological services in the epidemiological youth sample

Variables	HK-YES sample with 12-month MDE (n = 436)	
	Not receiving psychiatric/psychological services (n = 363)	Currently receiving psychiatric/psychological services (n = 73)
Background factors		
Female sex, n (%)	261 (71·9)	52 (71·2)
Age	20·21 (2·63)	19·59 (2·66)
Has childhood adversity, n (%)	203 (55·9)	52 (71·2)
Receiving government financial assistance, n (%)[a]	31 (8·6)	13 (18·1)
Not born in Hong Kong, n (%)	70 (19·3)	16 (21·9)
Psychological factors		
Resilience (CD-RISC-10)	20·96 (6·33)	16·22 (6·62)
Loneliness (UCLA-LS)	48·99 (8·93)	53·27 (9·76)
Digitalisation and lifestyle		
Smartphone overuse (CIAS-R ≥ 67), n (%)	166 (45·7)	30 (41·1)
Frequent nightmares (≥1 / week), n (%)	121 (33·3)	33 (45·2)
Days of regular physical activity (past week)	1·54 (1·49)	1·28 (1·23)
Family functioning		
Family dysfunction (BFRS)	22·98 (8·05)	24·90 (8·73)
Perceived stress and stressors		
≥2 personal SLEs, n (%)	120 (33·1)	27 (37·0)
≥2 social unrest-related TEs, n (%)	94 (25·9)	13 (17·8)
≥2 COVID-19 stressors, n (%)[c]	148 (43·8)	34 (50·7)

[a]Data on any government financial assistance received in this analysis were available from 434 participants.

[c]Data on COVID-19 stressors were collected since March 2020 and were available from 405 participants.

Note. Data are from 3030 participants of the HK-YES. Values are presented in the form of mean (SD), unless otherwise specified. BFRS = Brief Family Relationship Scale; CD-RISC-10 = Connor-Davidson Resilience Scale 10-Item; CIAS-R = Revised Chen Internet Addiction Scale; MDE = major depressive episode; SLEs = Stressful Life Events; UCLA-LS = 20-item UCLA Loneliness Scale.

Supplementary Table S12. Factors associated with current use of psychiatric/psychological services among young people with 12-month MDE in the epidemiological youth sample

Variables	HK-YES sample with 12-month MDE who are receiving psychiatric/psychological services (n = 73)	
	Univariate OR (95% CI)	*p*
**Background factors**		
Female sex	0·97 (0·56–1·69)	0·91
Age	0·92 (0·83–1·01)	0·069
Has childhood adversity	**1·95 (1·13–3·37)**	**0·017**
Receiving government financial assistance[a]	**2·35 (1·16–4·76)**	**0·017**
Not born in Hong Kong	1·18 (0·64–2·17)	0·61
**Psychological factors**		
Resilience (CD-RISC-10)	**0·88 (0·85–0·93)**	**<0·001**
Loneliness (UCLA-LS)	**1·05 (1·02–1·08)**	**<0·001**
**Digitalisation and lifestyle**		
Smartphone overuse (CIAS-R ≥ 67)	0·83 (0·50–1·38)	0·47
Frequent nightmares (≥1 / week)	1·65 (0·99–2·75)	0·054
Days of regular physical activity (past week)	0·88 (0·73–1·05)	0·16
**Family functioning**		
Family dysfunction (BFRS)	1·03 (1·00–1·06)	0·067
**Personal and population-level stressors**		
≥2 personal SLEs	1·19 (0·70–2·01)	0·66
≥2 social unrest-related TEs	0·62 (0·33–1·18)	0·52
≥2 COVID-19 stressors[c]	1·32 (0·78–2·24)	0·15

[a]Data on any government financial assistance received in this analysis were available from 434 participants.

Note. Data are from 3030 participants of the HK-YES. Statistics significant after accounting for multiple comparisons are boldfaced. BFRS = Brief Family Relationship Scale; CD-RISC-10 = Connor-Davidson Resilience Scale 10-Item; CIAS-R = Revised Chen Internet Addiction Scale; MDE = major depressive episode; PEs = COVID-19 pandemic-related events; PSQI = Pittsburgh Sleep Quality Index; SLEs = Stressful Life Events; TEs = social unrest-related traumatic events; UCLA-LS = 20-item UCLA Loneliness Scale.

Supplementary Table S13. Levels of functioning and quality of life among participants with 12-month MDE who are and are not currently receiving psychiatric/psychological services

Variables	HK-YES sample with 12-month MDE (n = 436)		
	Not receiving psychiatric/ psychological services (n = 363)	Currently receiving psychiatric/ psychological services (n = 73)	*p*
**Functioning**			
Social and occupational functioning (SOFAS)	**78·23 (7·73)**	**71·40 (9·01)**	**<0·001**
Days of reduced productivity	**4·81 (6·67)**	**9·10 (9·33)**	**<0·001**
Days of lost productivity	**1·20 (3·40)**	**3·81 (6·88)**	**<0·001**
**Health-related quality of life (QoL)**			
Overall health-related QoL (EQ-5D-5L)	**0·91 (0·10)**	**0·84 (0·13)**	**<0·001**
Physical health QoL (PCS-12)	**51·05 (8·12)**	**47·06 (8·06)**	**<0·001**
Mental health QoL (MCS-12)	**32·19 (11·57)**	**26·13 (11·53)**	**<0·001**
Global mental well-being (WHO-5)	**41·99 (17·71)**	**33·92 (17·87)**	**0·001**

Note. Values are presented in the form of mean (SD). EQ-5D-5L = EuroQol-5D; MDE = major depressive episode; QoL = quality of life; SF12–PCS = 12-Item Short Form Survey – Physical Health; SF12–MCS = 12-Item Short Form Survey – Mental Health; SOFAS = Social and Occupational Functioning Assessment Scale; WHO-5 = The World Health Organisation–Five Well-Being Index.

Reference

1. Svedberg P, Nygren JM, Staland-Nyman C, Nyholm M. The validity of socioeconomic status measures among adolescents based on self-reported information about parents occupations, FAS and perceived SES; implication for health related quality of life studies. BMC Med Res Methodol. 2016;16:48.
2. Currie C, Molcho M, Boyce W, Holstein B, Torsheim T, Richter M. Researching health inequalities in adolescents: the development of the Health Behaviour in School-Aged Children (HBSC) family affluence scale. Soc Sci Med. 2008;66(6):1429–36.
3. Walker ER, Druss BG. Cumulative burden of comorbid mental disorders, substance use disorders, chronic medical conditions, and poverty on health among adults in the U.S.A. Psychol Health Med. 2017;22(6):727–35.
4. Eaton WW, Muntaner C, Bovasso G, Smith C. Socioeconomic status and depressive syndrome: the role of inter- and intra-generational mobility, government assistance, and work environment. J Health Soc Behav. 2001;42(3):277–94.
5. Kessler RC, Ustün TB. The World Mental Health (WMH) Survey Initiative Version of the World Health Organization (WHO) Composite International Diagnostic Interview (CIDI). Int J Methods Psychiatr Res. 2004;13(2):93–121.
6. Campbell-Sills L, Stein MB. Psychometric analysis and refinement of the Connor-davidson Resilience Scale (CD-RISC): Validation of a 10-item measure of resilience. J Trauma Stress. 2007;20(6):1019–28.
7. Yu X, Zhang J. Factor analysis and psychometric evaluation of the Connor-Davidson Resilience Scale (CD-RISC) with Chinese people. Social Behavior and Personality: An International Journal. 2007;35(1):19–30.
8. She R, Yang X, Lau MM, Lau JT. Psychometric properties and normative data of the 10-item Connor–Davidson Resilience Scale among Chinese adolescent students in Hong Kong. Child Psychiatry Hum Dev. 2020;51:925–33.
9. Russell D, Peplau LA, Ferguson ML. Developing a measure of loneliness. J Pers Assess. 1978;42(3):290–4.
10. Cole A, Bond C, Qualter P, Maes M. A systematic review of the development and psychometric properties of loneliness measures for children and adolescents. Int J Environ Res Public Health. 2021;18(6):3285.
11. Hou T, Xie Y, Mao X, Liu Y, Zhang J, Wen J, Chen Y, Luo Z, Cai W. The mediating role of loneliness between social support and depressive symptoms among Chinese rural adolescents during COVID-19 outbreak: A comparative study between left-behind and non-left-behind students. Front Psychiatry. 2021;12:740094.
12. Mak KK, Lai CM, Ko CH, Chou C, Kim DI, Watanabe H, et al. Psychometric properties of the Revised Chen Internet Addiction Scale (CIAS-R) in Chinese adolescents. J Abnorm Child Psychol. 2014 Oct;42(7):1237–45.
13. Chen SH, Weng LJ, Su YJ, Wu HM, Yang PF. Development of Chinese Internet Addiction Scale and its psychometric study. Chinese Journal of Psychology. 2003;45(3):279–94.
14. Ko CH, Yen JY, Chen SH, Yang MJ, Lin HC, Yen CF. Proposed diagnostic criteria and the screening and diagnosing tool of Internet addiction in college students. Compr Psychiatry. 2009;50(4):378–84.
15. Wong SMY, Chen EYH, Wong CSM, Suen YN, Chan DLK, Tsang SH, et al. Impact of smartphone overuse on 1-year severe depressive symptoms and momentary negative affect: Longitudinal and experience sampling findings from a representative epidemiological youth sample in Hong Kong. Psychiatry Res. 2022 Nov 2;318:114939.
16. Guo S, Sun W, Liu C, Wu S. Structural Validity of the Pittsburgh Sleep Quality Index in Chinese Undergraduate Students. Front Psychol. 2016;7:1126.
17. Buysse DJ, Reynolds CF 3rd, Monk TH, Berman SR, Kupfer DJ. The Pittsburgh Sleep Quality Index: a new instrument for psychiatric practice and research. Psychiatry Res. 1989;28(2):193–213.
18. Li SX, Zhang B, Li AM, Wing YK. Prevalence and correlates of frequent nightmares: a community-based 2-phase study. Sleep. 2010;33(6):774–80.
19. Janson C, Gislason T, De Backer W, Plaschke P, Björnsson E, Hetta J, et al. Prevalence of sleep disturbances among young adults in three European countries. Sleep. 1995;18(7):589–97.
20. Paul F, Schredl M, Alpers GW. Nightmares affect the experience of sleep quality but not sleep architecture: an ambulatory polysomnographic study. Borderline Personal Disord Emot Dysregul. 2015;2:3.
21. Wong SMY, Hui CLM, Cheung VKW, Suen YN, Chan SKW, Lee EHM, et al. Prevalence of frequent nightmares and their prospective associations with 1-year psychiatric symptoms and disorders and functioning in young adults: a large-scale epidemiological study in Hong Kong. Sleep. 2022.
22. Craig CL, Marshall AL, Sjöström M, Bauman AE, Booth ML, Ainsworth BE, et al. International physical activity questionnaire: 12-country reliability and validity. Med Sci Sports Exerc. 2003;35(8):1381–95.
23. Macfarlane DJ, Lee CCY, Ho EYK, Chan KL, Chan DTS. Reliability and validity of the Chinese version of IPAQ (short, last 7 days). J Sci Med Sport. 2007;10(1):45–51.
24. Fok CCT, Allen J, Henry D, People Awakening Team. The brief family relationship scale: a brief measure of the relationship dimension in family functioning. Assessment. 2014;21(1):67–72.
25. Brugha T, Bebbington P, Tennant C, Hurry J. The List of Threatening Experiences: a subset of 12 life event categories with considerable long-term contextual threat. Psychol Med. 1985;15(1):189–94.
26. Wong SMY, Hui CLM, Wong CSM, Suen YN, Chan SKW, Lee EHM, et al. Mental Health Risks after Repeated Exposure to Multiple Stressful Events during Ongoing Social Unrest and Pandemic in Hong Kong: The Role of Rumination: Risques pour la santé mentale après une exposition répétée à de multiples événements stressants d'agitation sociale durable et de pandémie à Hong Kong: le rôle de la rumination. Can J Psychiatry. 2021;66(6):577–85.

27. Wong SMY, Hui CLM, Suen YN, Wong CSM, Chan SKW, Lee EHM, et al. The impact of social unrest and pandemic on mental health of young people in Hong Kong: The transdiagnostic role of event-based rumination. Aust N Z J Psychiatry. 2021;56(4):376–84.
28. Wong SMY, Ip CH, Hui CLM, Suen YN, Wong CSM, Chang WC, et al. Prevalence and correlates of suicidal behaviours in a representative epidemiological youth sample in Hong Kong: the significance of suicide-related rumination, family functioning, and ongoing population-level stressors. Psychol Med. 2022;1–11.
